# Impact of EMG-based interfaces for hand motor rehabilitation in stroke: A systematic review with evidence gap map and meta-analysis

**DOI:** 10.1007/s11517-026-03660-7

**Published:** 2026-08-28

**Authors:** Cristian D. Guerrero-Mendez, Natanael Pereira Batista, José Osmar Alves Filho, Carina Marconi Germer, Leonardo Abdala Elias

**Affiliations:** 1 Department of Electronics and Biomedical Engineering, School of Electrical and Computer Engineering, University of Campinas - UNICAMP, Campinas, 13083-852 SP Brazil; 2 Neural Engineering Research Laboratory, Center for Biomedical Engineering, University of Campinas - UNICAMP, Campinas, 13083-881 SP Brazil; 3 Department of Physical Therapy, Faculdade Israelita de Ciências da Saúde Albert Einstein, Hospital Israelita Albert Einstein, São Paulo, Brazil

**Keywords:** EMG-based intervention, Stroke rehabilitation, Hand, Wrist, Meta-analysis

## Abstract

Electromyography (EMG)-based interventions have been proposed to improve hand/wrist motor recovery after stroke; however, their clinical value remains uncertain, as evidence for their effectiveness is inconsistent. This systematic review with meta-analysis evaluated the impact of different EMG-based rehabilitation approaches compared with non-EMG-based interventions in individuals with stroke. Seven databases were searched from inception to November 17, 2025, in accordance with PRISMA guidelines. Randomized controlled trials comparing EMG-based and non-EMG-based interventions for post-stroke hand or wrist rehabilitation were eligible. Interventions were classified into four categories: EMG-triggered electrical stimulation (EMG-ES), EMG-ES combined with other interventions, EMG-based robotic platforms, and EMG biofeedback combined with other interventions. Risk of bias was assessed using the Cochrane Risk of Bias 2 tool, and certainty of evidence was evaluated using the Grading of Recommendations Assessment, Development and Evaluation (GRADE) approach. Meta-analyses were conducted for clinical outcomes classified according to the International Classification of Functioning, Disability and Health (ICF) domains at post-intervention, follow-up <3 months, and follow-up ≥3 months. Thirty-eight studies involving 1,132 participants were included, of which 31 contributed to the meta-analyses. Eight studies were judged to have a low risk of bias, 13 to have some concerns, and 17 to have a high risk of bias. EMG-ES alone was not superior to conventional therapy. In contrast, EMG-ES combined with other interventions produced greater improvements across all ICF domains. EMG-based robotic platforms reduced wrist and finger spasticity, with effects sustained at long-term follow-up. EMG biofeedback primarily improved outcomes within the ICF body functions and structures domain. The effectiveness of EMG-based interventions depends on the rehabilitation strategy. EMG-ES, combined with other therapies, may be prioritized, whereas EMG-based robotic platforms may be useful in reducing spasticity. Further high-quality studies are needed to establish the clinical effectiveness of EMG-biofeedback approaches.

**Graphical abstract:**

The graphical abstract summarizes the findings of a systematic review and meta-analysis including 38 studies and 1,132 participants. Four types of sEMG-based rehabilitation interfaces were examined across the International Classification of Functioning, Disability and Health (ICF) domains of Body Functions and Structures, Activity, Participation, and Patient-reported outcomes. The symbols indicate the direction of the observed effects and the certainty of the evidence according to GRADE. The results suggest that the effects of sEMG-based rehabilitation vary according to the type of interface. The combination of sEMG-triggered electrical stimulation with other interventions produced the most consistent improvements across the ICF domains. In contrast, sEMG-triggered stimulation alone and sEMG-biofeedback combined with other interventions showed fewer and less consistent benefits. sEMG-based robotic platforms also showed potential, particularly for reducing spasticity. Together, these findings favor the use of combined sEMG-triggered interventions while supporting further investigation of sEMG-based robotic approaches in rehabilitation.

**Figure:**
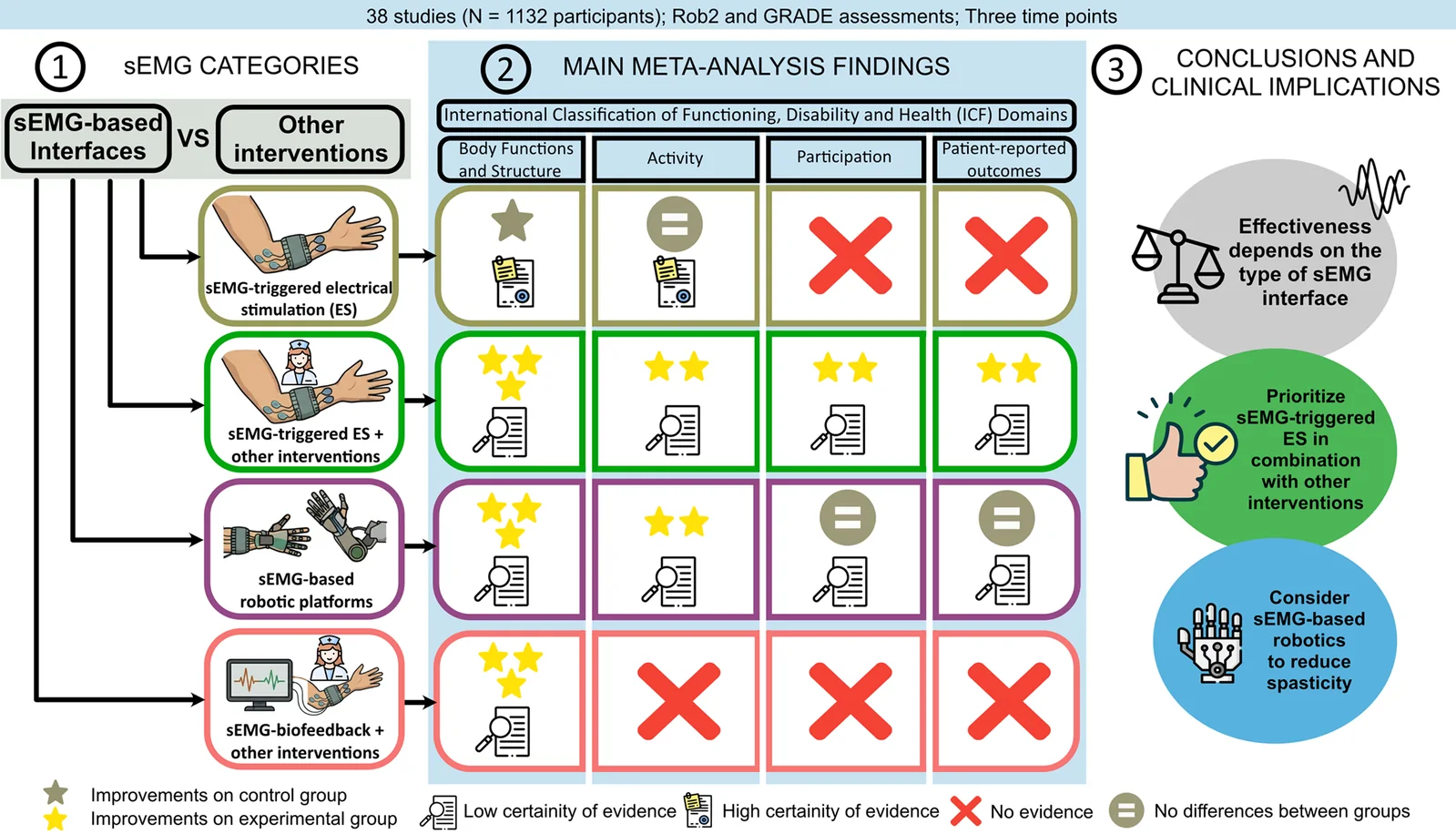

**Supplementary Information:**

The online version contains supplementary material available at https://doi.org/10.1007/s11517-026-03660-7.

## Introduction

Stroke is one of the leading causes of disability worldwide, frequently resulting in partial or severe motor impairments [1]. The upper limbs are among the most commonly affected body segments, with approximately 50% to 80% of stroke survivors presenting some degree of upper-limb dysfunction [2]. Consequently, hemiparesis represents the most prevalent sequela following stroke, significantly limiting the performance of activities of daily living and restricting participation in social and physical environments [3]. These motor impairments highlight the critical need for effective rehabilitation strategies to restore upper-limb function. This need is of paramount importance for hand/wrist movements, since these segments are essential for fine, highly controlled actions and, therefore, for functional independence [4]. However, despite the growing interest in technology-assisted rehabilitation, the effectiveness of technological interventions compared with conventional rehabilitation approaches remains unclear.

Electromyography (EMG)-based clinical interventions have been proposed as an alternative approach for motor rehabilitation after stroke. These interventions have been used to control electrical stimulation (ES) [5], implement combined approaches [6], operate powered robotic platforms [7], and provide biofeedback [8]. EMG-based therapies have gained increasing attention in neurorehabilitation because they allow the detection of residual muscle activity and provide signals that can be used for real-time control of assistive systems [9]. Unlike conventional approaches, which mainly rely on therapist-guided exercises, EMG-based approaches enable greater patient engagement and integrate voluntary muscle activation into the therapeutic process [10]. This may facilitate motor recovery and improve functional outcomes.

Despite these potential advantages, the evidence regarding their effectiveness remains inconclusive. Some systematic reviews have reported that EMG-based interventions—such as EMG-triggered ES and EMG-based robotic systems—produce greater improvements in hand, wrist, and upper-limb motor function compared with conventional ES or rehabilitation approaches [11, 12]. However, a more recent systematic review with meta-analyses did not find consistent evidence supporting the superiority of EMG-based interventions over non-EMG-based therapies and was unable to determine which EMG-based intervention is most effective [13]. These discrepancies may be partly explained by methodological limitations in previous reviews, such as the low number of included studies, the lack of systematic categorization of the interventions, the restriction to use only one type of intervention, the fragmentation of the meta-analyses, the lack of multiple clinical outcomes and domain segmentation, and the combination of different clinical outcomes to perform the meta-analyses. The above reflects the important methodological and clinical limitations in the existing literature. As a result, potentially relevant effects of EMG-based interfaces on distal upper-limb segments may have been masked.

In this context, a systematic review and meta-analysis focused on hand/wrist outcomes is necessary to clarify the clinical relevance of EMG-based interventions and to identify approaches that may offer meaningful benefits for motor recovery following stroke. Thus, this systematic review and meta-analysis aim to evaluate the impact of EMG-driven hand/wrist motor rehabilitation approaches compared with non-EMG-based rehabilitation strategies in individuals with stroke, and to identify which types of EMG-based interventions may be clinically beneficial for specific clinical outcomes. This review responds to the following question: What is the impact of hand/wrist rehabilitation using EMG signals as an interface compared to conventional therapy or non-EMG-based intervention for people with stroke?

## Methods

### Registration and protocol

This systematic review has been registered in an international database of systematic reviews in health and social care, PROSPERO (https://www.crd.york.ac.uk/prospero/; registration number: CRD420251089447), without deviation from the protocol, thereby providing greater transparency and accountability [14]. The current systematic review follows the Preferred Reporting Items for Systematic Reviews and Meta-Analyses (PRISMA) guidelines [15]. This study did not require ethical approval as it involved a thorough analysis of previously published data.

### Literature search strategy

An extensive literature search was conducted using seven datasets available through the University of Campinas (UNICAMP) library system: Web of Science (WOS), Scopus, Excerpta Medica Database (EMBASE), Medical Literature Analysis and Retrieval System Online (MEDLINE - PubMed), Database of Abstracts of Reviews of Effects (DARE), Disability and Rehabilitation Literature Database (REHABDATA), Psychological Information Database (PsycINFO). These databases were searched from their inception until November 17, 2025. The search strategy combined free-text keywords and Medical Subject Headings (MeSH) related to stroke, EMG-based interventions, hand and wrist rehabilitation, and conventional therapy. The search was applied to the title, abstract, and keywords fields, with restrictions on language and publication type (only full-text articles published in English and journals). The “OR” and “AND” boolean operators were used to combine the keywords. The overall search strategy included terms related to stroke, EMG, and hand function recovery. The detailed search strategy can be found in **Supplementary Material **1.

One author (CDGM) imported all identified studies to Rayyan (a web platform for systematic review) [16], where duplicates were automatically identified using the Rayyan AI system, and manually removed by CDGM. Finally, a hand-searching strategy was conducted to identify additional studies that were not captured by the search strategy. The reference lists of all included studies were manually examined for potentially relevant articles.

### Eligibility criteria

Studies were eligible for inclusion if they met the following criteria: 1. involved participants with stroke (older than 18 years of age); 2. evaluated hand or finger motor rehabilitation using functional outcome assessments; 3. investigated EMG-based clinical interventions; 4. employed a randomized controlled trial design or any level of randomization; and 5. were published as full-text articles in English.

Studies were excluded if they met any of the following criteria: 1. conference abstracts or proceedings, case reports, or review articles; 2. absence of functional outcome assessments; 3. inclusion of healthy participants as control group; 4. use of invasive electrodes; 5. absence of hand or finger motor rehabilitation; 6. evaluation of EMG-based systems without implementation of an EMG-based intervention; 7. focus on lower-limb rehabilitation; 8. interventions involving spinal or supraspinal activation techniques (e.g., transcranial magnetic stimulation, transcranial direct current stimulation, or electroencephalography); or 9. absence of a conventional therapy or non-EMG-based intervention comparison group.

One author (CDGM) performed the title and abstract screening. Subsequently, full-text screening was conducted independently by two authors (CDGM, NPB, or JOAF). In cases of disagreement, a third reviewer (among the three authors mentioned above) was consulted to resolve discrepancies and reach consensus. The UNICAMP’s library purchased articles with subscription access for full-text screening.

### Risk of bias assessment

The risk of bias was assessed using the Revised Cochrane Risk of Bias 2 tool for randomized trials (RoB2) [17, 18] by two independent reviewers (among CDGM, NPB, and JOAF). The RoB2 tool evaluates the risk of bias across five domains: the randomization process, deviations from the intended interventions, missing outcome data, measurement of outcomes, and selective reporting of results [17, 18]. The risk of bias for each domain was classified as *low risk*, *some concerns*, or *high risk*. Overall, the risk of bias was rated as “low risk” when all domains were judged “low risk”. A study was rated as having “some concerns” when at least one domain was judged “some concerns” and none were judged “high risk”. A study was rated as “high risk” when at least one domain was judged “high risk”, or when three or more domains were judged as “some concerns”. Disagreements were resolved through discussion among the authors to reach consensus, and a third reviewer was consulted when consensus could not be reached. Finally, differences across raters were assessed by calculating agreement percentages.

### Data extraction

Data were extracted using a standardized data-extraction Microsoft Excel sheet. The following information was collected: study characteristics (authors and year of publication); participant characteristics (population, age, time since stroke, stroke stage, and number of participants in each group); intervention characteristics (number of sessions per week, total number of sessions and intervention duration, and intervention duration); type of intervention in both experimental and control groups; clinical outcomes/measures (mean ± standard deviation for both experimental and control groups); timing of outcome assessment (immediately post-intervention or follow-up); and study conclusions. Data extraction was performed independently by two reviewers (CDGM, NPB, or JOAF). Disagreements were resolved through discussion, and if consensus could not be reached, a third reviewer was involved.

Clinical outcome data were extracted to evaluate immediate (post-intervention) and follow-up effects, which were categorized into two time ranges: *< 3* months and *≥ 3* months. We defined stroke stage as acute (*< 3* months), subacute (*≥ 3* and *< 6* months), and chronic (*≥ 6* months). Finally, when numerical raw data were not provided, data were extracted from figures using a digitizing program (PlotDigitizer, Version 3.0; available at https://plotdigitizer.com/).

### Data analysis

The included studies were pooled in a meta-analysis using the subscription version of Review Manager (RevMan, The Cochrane Collaboration, Copenhagen, DK). Considering the aim of this review, meta-analyses were grouped according to the type of intervention, which was categorized as follows:

- **EMG-triggered ES (EMG-ES):** Interventions in which myoelectric activity from a target muscle triggers the application of ES once a predefined activation threshold is exceeded [19].
- **EMG-ES combined with other interventions:** Interventions integrating EMG-triggered ES with additional rehabilitation approaches, such as conventional therapy, mirror therapy, task-oriented training, or motor imagery, to increase practice intensity and engage complementary mechanisms of motor recovery [10].
- **EMG-based robotic platforms:** Interventions in which EMG signals are used to estimate motor intention to control robotic devices that provide movement assistance during the rehabilitation process [20].
- **EMG-based biofeedback combined with other interventions:** Interventions that convert EMG activity into real-time feedback (e.g., visual or auditory) to the patient, combined with additional therapeutic approaches (e.g., conventional therapy or ES), to facilitate motor learning or to promote sensorimotor reorganization [21].

These categories were evaluated against conventional rehabilitation or non-EMG-based interventions. Different subgroups within each meta-analysis category were defined according to the specific intervention and/or comparator to analyze the effects of individual interventions.

For the meta-analyses, we extracted the mean and standard deviation (SD) of the change scores (i.e., post- minus pre-intervention) for each intervention group. When change scores were not directly reported but means and SDs were available at each time point, the mean and SD of the change were manually calculated in accordance with the Cochrane Handbook [22]. Because calculation of the SD of change requires an outcome-specific correlation coefficient, these coefficients were derived for clinical outcomes using data from comparable included studies that reported baseline, post-intervention, and change SDs. For outcomes in which the correlation coefficient could not be estimated due to insufficient data (e.g., missing SD of change), a conservative value of 0.5 was assumed. Additionally, when change scores were reported as means with 95% confidence intervals (CIs) or as medians with interquartile ranges, these data were converted to mean ± SD using established methods, following the recommendations of the Cochrane Handbook for Systematic Reviews of Interventions (Chapter 6) [22] and previous literature [23]. When insufficient data were provided, corresponding authors were contacted to request the missing information.

Clinical outcomes were analyzed separately in each categorized meta-analysis described above, pooling only studies that used the same clinical assessment. These clinical assessments were classified according to the ICF domains [24, 25] (Details in **Supplementary Material **2). Studies for which outcome data could not be obtained after contacting the corresponding authors were excluded from the meta-analyses but remained included in the qualitative (descriptive) review. According to the Cochrane Handbook for Systematic Reviews of Interventions, Chapter 6 [22], when a study included multiple intervention groups relevant to the same comparison, these groups were combined into a single intervention group (experimental or control) to avoid unit-of-analysis errors and double counting in the meta-analysis.

As each meta-analysis focused on a single clinical outcome, score transformations to harmonize the direction of improvement (higher versus lower scores) were not required. Overall, effect sizes included data from studies involving participants in the acute, subacute, and chronic stages of stroke. Effect sizes were calculated by entering the mean change values, SD change values, and sample sizes for each group into RevMan, which computed the standardized mean differences (SMDs) with 95% CIs using a fixed-effect model. The random-effect model was used for meta-analysis, including variables with possibly different units, such as grip strength measurements, using the restricted maximum-likelihood heterogeneity estimator. Heterogeneity was reported using the $I^2$ statistic.

The magnitude of the SMDs was classified as small for values *< 0.59*, moderate for values between 0.60 and 1.19, and large for values *≥ 1.20* [26]. Group differences were considered statistically significant at *p ≤ 0.05*. Finally, sensitivity analyses were conducted for meta-analyses that included studies with two or more evaluation time points falling within the follow-up ranges. If there were no changes in results in the sensitivity analysis, the time point closest to the upper boundary of the range was selected for that study; specifically, for the *< 3*-month range, the assessment closest to 3 months was used, and for the *≥ 3*-month range, the assessment furthest beyond 3 months was considered.

### Level of evidence assessment

The certainty of evidence for each meta-analysis was assessed using the updated version of the Grading of Recommendations Assessment, Development and Evaluation (GRADE) approach [27]. The criteria comprise five domains: risk of bias, imprecision, inconsistency, indirectness and publication bias. A detailed description for each domain is available in **Supplementary Material **3.

Two independent authors (CDGM and NPB) assessed the certainty of the evidence, with disagreements resolved by consensus. Certainty of evidence was classified as high, moderate, low, or very low according to the GRADE guidelines. Each meta-analysis initially started at high certainty and was downgraded by one level when concerns were identified in any GRADE domain, and upgraded when a large magnitude effect was observed.

### Evidence gap map

An evidence gap map was created to provide an overview of the available evidence on EMG-based interventions for hand/wrist motor rehabilitation after stroke. This map facilitates the identification of clinical outcomes with sufficient or insufficient evidence based on the number of comparable studies [28], and highlights areas where evidence is lacking and where this review cannot draw conclusions. For this purpose, all investigations within the same intervention categories and clinical outcomes (each meta-analysis performed) were combined with build the evidence gap map. The intervention categories were the same as those used in meta-analyses. A summary of the meta-analysis outcomes was also added to identify differences in direction (respective SMD strengths) and certainty of evidence (GRADE scores).

**Fig. 1:**
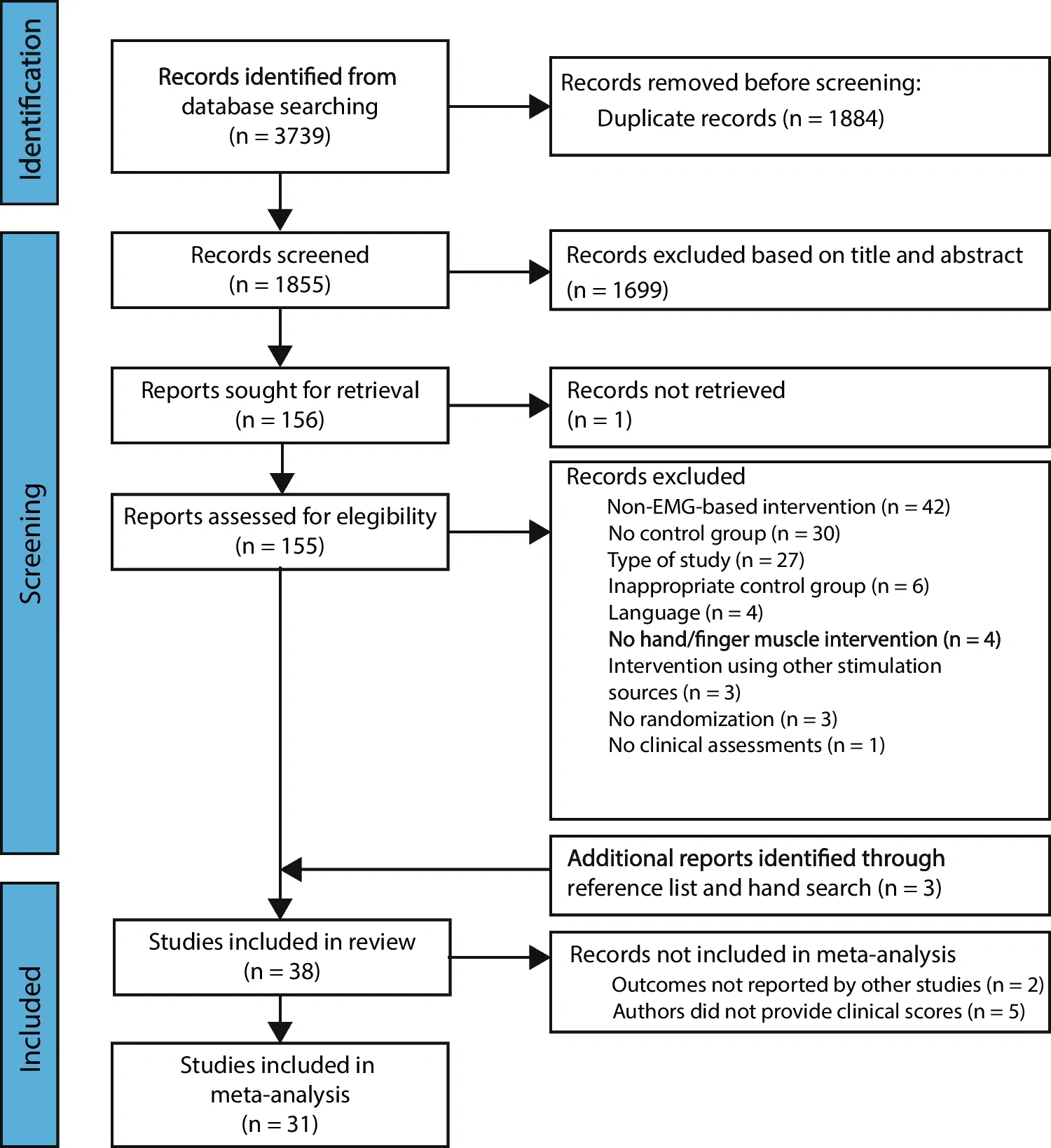
PRISMA flow-chart diagram of the screening process

## Results

### Study selection

In total, 1,855 studies were identified after duplicate removal. Following the screening, 35 studies met the inclusion criteria [5–8, 19, 29–58]. Three additional studies were identified through reference list screening and hand searching [59–61], resulting in 38 studies included in the review (see Fig. 1). During full-text screening, one study could not be retrieved [62]. From the retrieved full-text articles, 120 studies were excluded; the specific references and reasons for exclusion are detailed in **Supplementary Material **4. Seven studies were excluded from the meta-analysis. From these, two studies reported outcomes not included in other studies [8, 58], and in five [30, 44, 47, 49, 57] clinical scores could not be obtained despite contacting the authors. Details of the main findings from these studies are provided in **Supplementary Material **5.

### Study characteristics

The characteristics of the included studies are presented in **Supplementary Material **6. In total, the studies involved 1,132 individuals with stroke, with sample sizes ranging from nine participants [54] to 101 participants [19] with a mean of 29.78 ± 22.87 participants. Most studies (n = 16) conducted clinical interventions in individuals with chronic stroke [32, 35, 37, 39, 42–45, 47, 49, 53, 55–57, 59, 61]. Nine studies focused on subacute stroke [5, 29, 34, 36, 38, 41, 46, 48, 51], six on acute stroke [6, 7, 19, 52, 54, 58], and seven included mixed populations (acute/subacute or chronic stroke patients) [8, 30, 31, 33, 40, 50, 60]. The mean age of participants ranged from 42.60 years [31] to 75.58 years [32] with a mean of 59.54 ± 6.23 years. Only one study did not report participants’ ages [46].

Across the included studies, the number of intervention sessions ranged from 4 [44] to 126 [42], with a mean of 34.19 ± 23.03 sessions. The intervention frequency varied from 2 [37, 55] to 21 sessions per week [42], with a mean of 6.67 ± 3.67 sessions per week. The total intervention duration ranged from 2 weeks [8, 44, 57] to 52 weeks [37], with a mean duration of 6.66 ± 8.83 weeks. Only one study tailored the treatment protocol according to individual participant needs [54]. Based on the included studies, four main categories of EMG-based interventions were identified. Six studies were classified exclusively as EMG-ES [5, 19, 31, 42, 49, 51], 21 were classified as EMG-ES combined with other interventions [6, 29, 30, 32, 33, 38, 39, 41, 43–46, 48, 50, 52–54, 57–60], six as EMG-based robotic platforms [7, 34, 35, 55, 56, 61], and five as EMG-biofeedback combined with other interventions [8, 36, 37, 40, 47]. The control intervention included conventional rehabilitation, passive and robot-assisted movements, task-oriented rehabilitation, placebo stimulation and biofeedback, conventional stimulation, conventional mirror therapy, physical training, low-intensity physical activities, and combinations of some of these.

Several outcome measures were used to assess the domains of *body functions and structures*, *activity*, *participation*, and *patient-reported outcomes*. The following clinical outcomes were found: Action Research Arm Test (ARAT); Fugl–Meyer Assessment for Upper Extremity (FMA-UE) and subscales; Brunnstrom scores; Motricity Index; Motor Status Scale (MSS); Chedoke-McMaster Stroke Assessment (CMSA-Hand); Functional Independence Measure (FIM); Barthel Index (BI); Manual Function Test (MFT); Grip and pinch strength; Manual Muscle Testing (MMT); Modified Ashworth Scale (MAS); Motor Activity Log – Amount of Use (MAL-AOU); Motor Activity Log – Quality of Movement (MAL-QOM); Box and Blocks Test (BBT); Range of Motion (ROM); Wolf Motor Function Test-time (WMFT-Time) and WMFT - Functional Ability Scale (WMFT - FAS); Stroke Impact Scale (SIS); Nine Hole Peg Test (NHPT); Arm Motor Ability Test (AMAT); Upper Extremity Function Test (UEFT); General Jebsen–Taylor Hand Function Test (JTHFT); Minnesota Manual Dexterity Test (MMDT); Reaching Performance Scale (RPS); Stroke-Specific Quality of Life Scale (SS-QOL); Disability of the Arm, Shoulder and Hand questionnaire (DASH); and Individually Prioritized Problem Assessment (IPPA).

Most studies evaluated more than one domain using the implemented clinical outcomes. Four studies assessed only the *body functions and structures* domain [36, 41, 52, 58], while two studies evaluated only the *activity* domain [8, 44]. All included studies conducted clinical evaluations at baseline and immediately after the intervention. Six studies included follow-up assessments shorter than 3 months [42, 51, 55, 56, 58, 60], while six studies reported follow-up assessments of 3 months or longer [29–31, 34, 48, 61]. Two studies conducted follow-up evaluations in both time ranges (i.e., shorter and longer than 3 months) [5, 19].

### Quality assessment

**Fig. 2:**
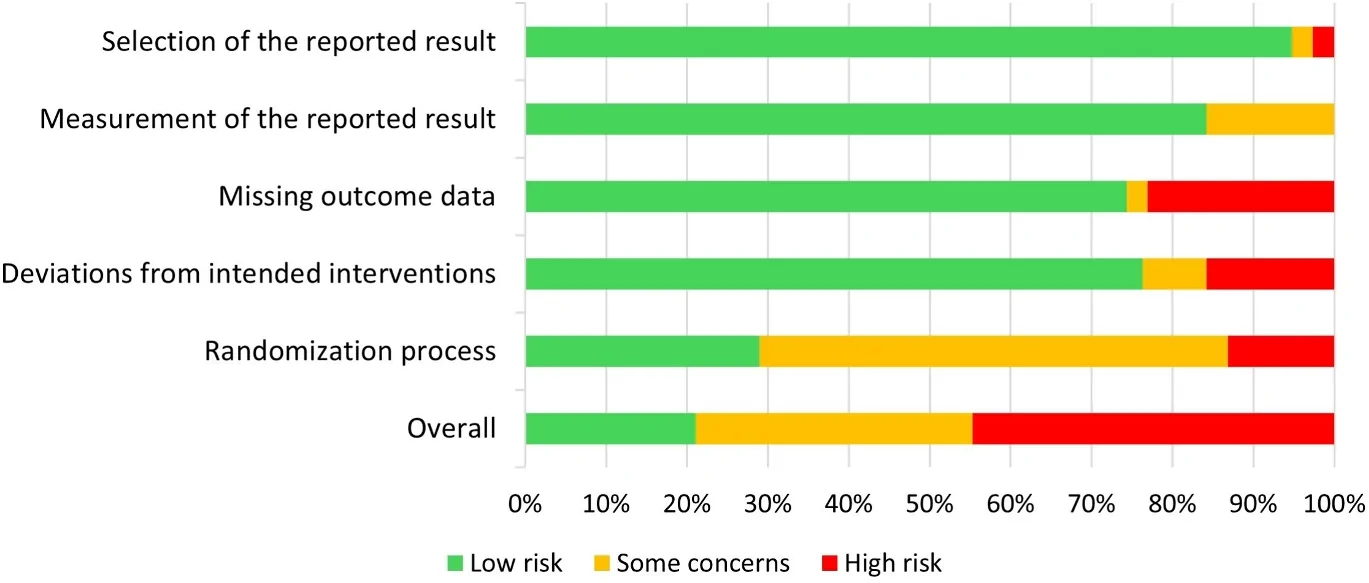
Risk of bias of the included studies

The methodological quality of the included studies, assessed using the RoB 2 tool, indicated that eight studies were classified as having a low risk of bias [5, 19, 36, 38, 50, 53, 55, 58], 13 as having some concerns [6, 8, 32–34, 37, 39, 40, 42, 48, 56, 60, 61], and 17 as having a high risk of bias [7, 29–31, 35, 41, 43–47, 49, 51, 52, 54, 57, 59]. The relative proportions of risk-of-bias judgments for each domain and the overall RoB 2 evaluation are presented in Fig. 2. Detailed assessments for each study are provided in **Supplementary Material **7. The GRADE assessment of the meta-analyses indicated certainty of evidence ranging from very low to high, which will be presented with the meta-analyses.

### Meta-Analysis of EMG-based intervention

Results from the meta-analysis are organized by intervention type and presented within the ICF domains.

#### Effects of EMG-ES

**Fig. 3:**
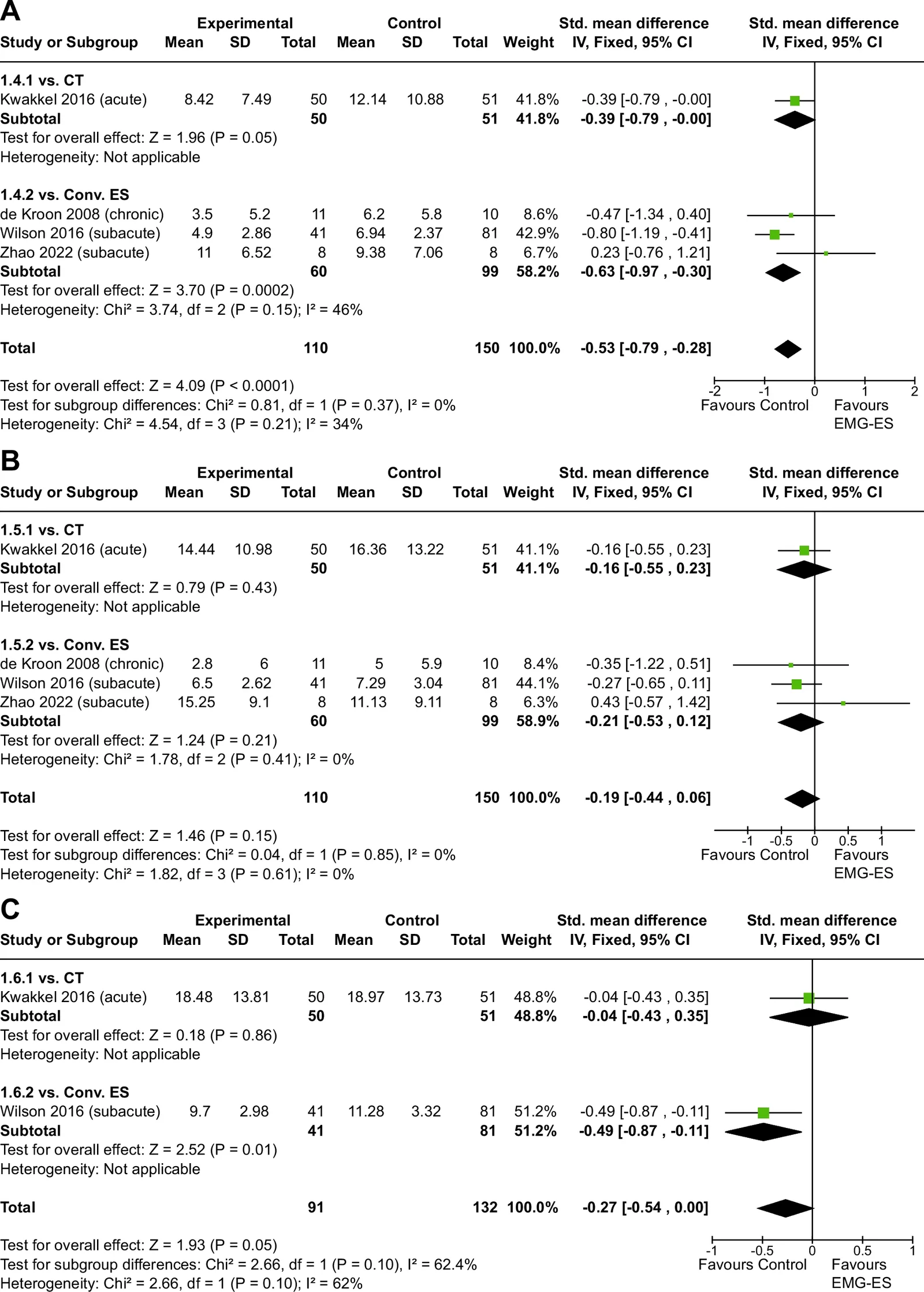
Meta-Analysis of the effects of EMG-ES versus control interventions on the *body functions and structures* domain of the International Classification of Functioning, Disability and Health (ICF), assessed using Fugl-Meyer Assessment – Upper Extremity (FMA-UE) immediately after intervention (**A**), at follow-up < 3 months (**B**), and at follow-up *≥ * 3 months (**C**). Abbreviations: CT, conventional therapy; ES, electrical stimulation

**Fig. 4:**
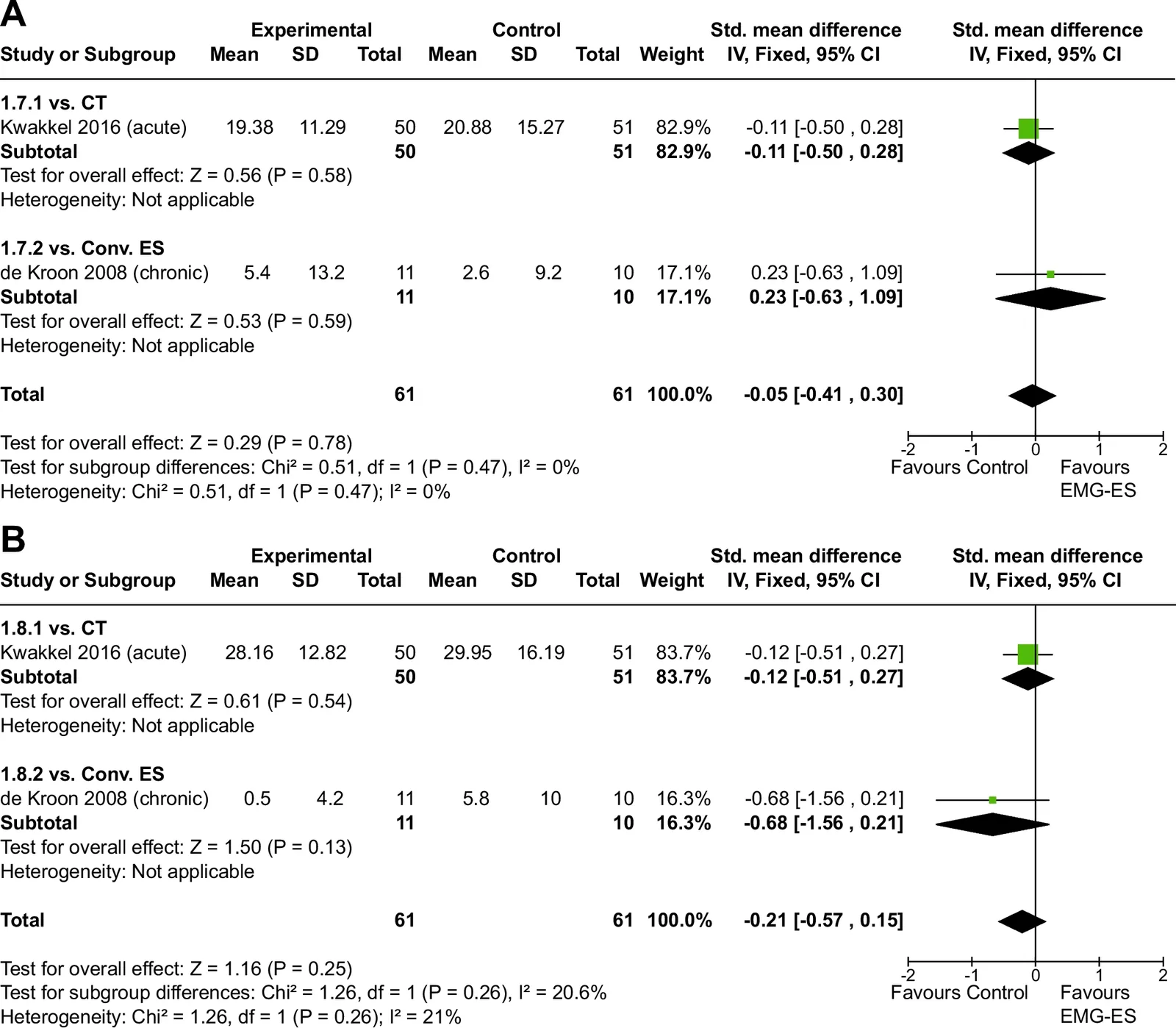
Meta-Analysis of the effects of EMG-ES versus control interventions on the *body functions and structures* domain of the International Classification of Functioning, Disability and Health (ICF), assessed using Motricity Index immediately after intervention (**A**), and at follow-up < 3 months (**B**). Abbreviations: CT, conventional therapy; ES, electrical stimulation

**Fig. 5:**
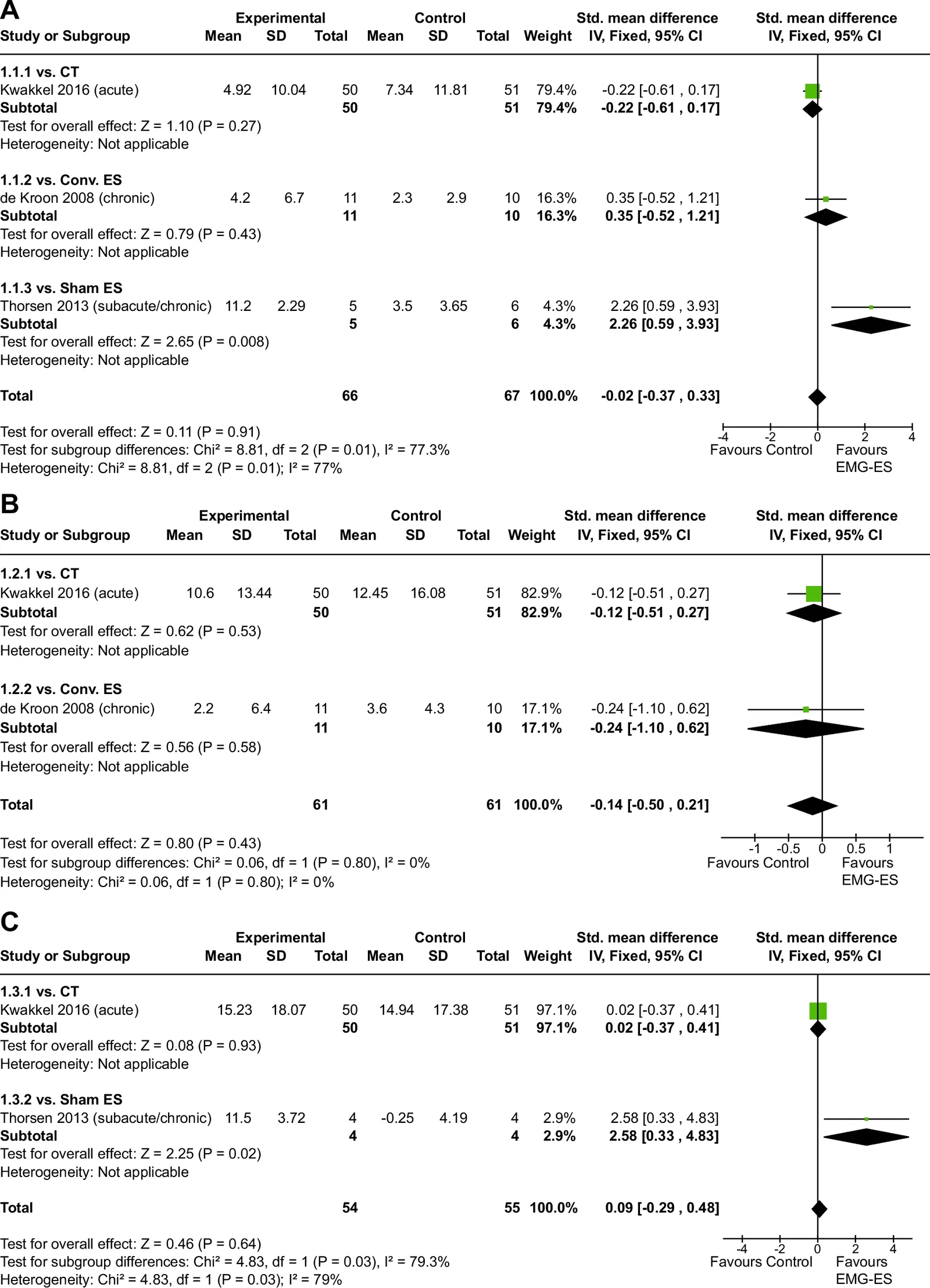
Meta-Analysis of the effects of EMG-ES versus control interventions on the *activity* domain of the International Classification of Functioning, Disability and Health (ICF), assessed using Action Research Arm Test (ARAT) immediately after intervention (**A**), at follow-up < 3 months (**B**), and at follow-up *≥ * 3 months (**C**). Abbreviations: CT, conventional therapy; ES, electrical stimulation

Figures 3, 4, and 5 present the meta-analyses conducted when the experimental group intervention was based exclusively on EMG-ES. In this category of interventions, the meta-analyses were stratified by comparator type (i.e., control): conventional ES, conventional therapy, and sham stimulation. It is important to highlight that sensitivity analyses performed with studies that included different time-point assessments across the two types of follow-up [5, 19] did not reveal any significant differences from the original results.

##### Body functions and structures

Low certainty evidence indicated greater improvements in FMA-UE immediately after the intervention in the control intervention (small SMD = −0.53, 95% CI = −0.79 to −0.28, Fig. 3A). Moderate and high certainty evidence indicated no difference between interventions in FMA-UE at follow-up < 3 months (SMD = −0.19, 95% CI = −0.44 to 0.06, Fig. 3B) and at follow-up *≥ * 3 months (SMD = −0.27, 95% CI = −0.54 to −0.00; Fig. 3C), respectively. Moderate certainty evidence indicated no difference between interventions for Motricity Index immediately after the intervention (SMD = −0.05, 95% CI = −0.41 to 0.30; Fig. 4A) and at follow-up < 3 months (SMD = −0.21, 95% CI = −0.57 to 0.15; Fig. 4B).

##### Activity

Low to moderate certainty evidence indicated no difference between interventions in ARAT scores immediately after the intervention (SMD = −0.02, 95% CI = −0.37 to 0.33, Fig. 5A), at follow-up < 3 months (SMD = −0.14, 95% CI = −0.50 to 0.21, Fig. 5B), and at follow-up *≥ * 3 months (SMD = 0.09, 95% CI = −0.29 to 0.48; Fig. 5C).

#### Effects of EMG-ES combined with other interventions

**Fig. 6:**
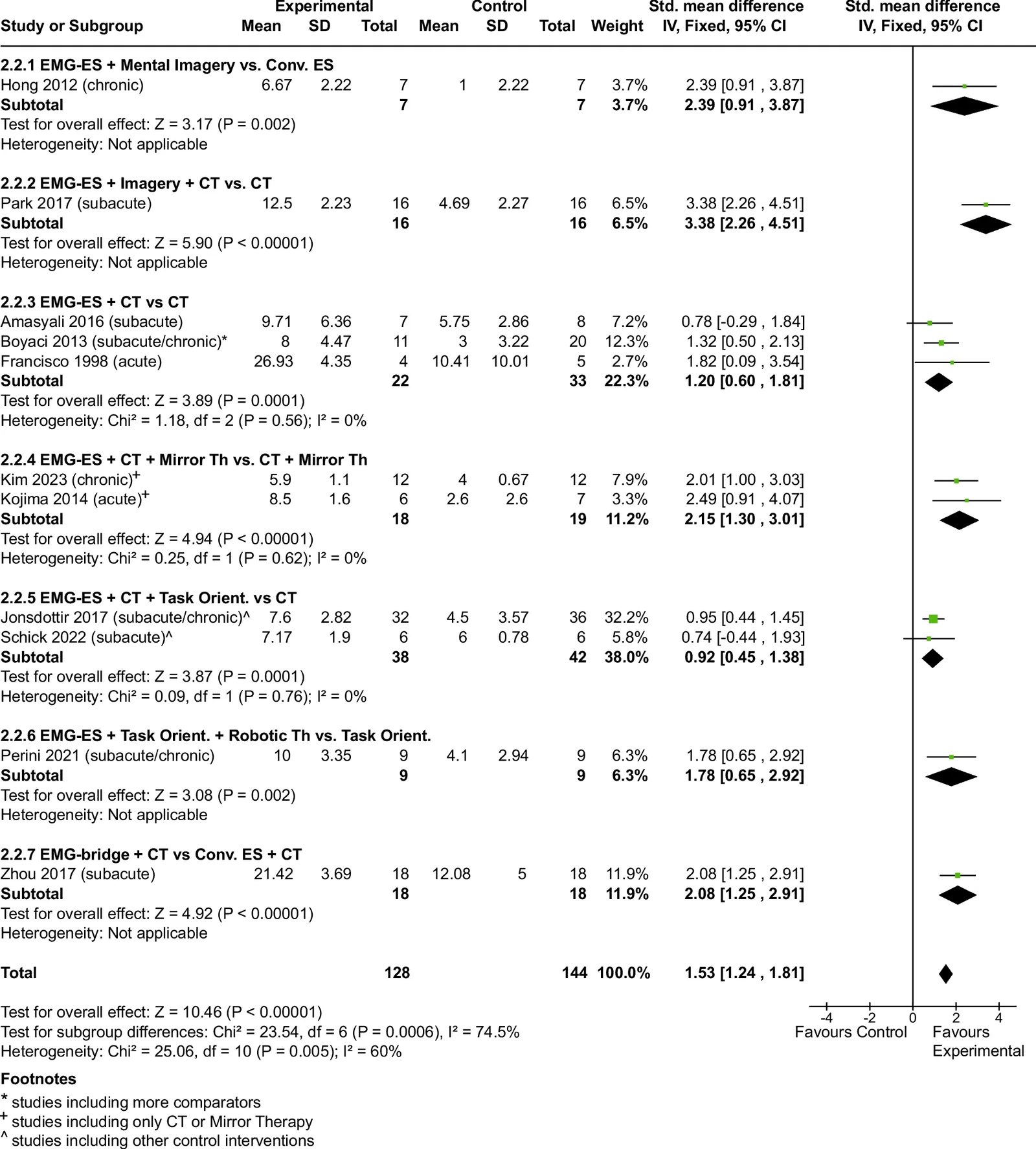
Meta-Analysis of the effects of EMG-ES combined with other interventions versus the comparators on the *body functions and structures* domain of the International Classification of Functioning, Disability and Health (ICF), assessed using Fugl-Meyer Assessment – Upper Extremity (FMA-UE) immediately after intervention. Abbreviations: CT, conventional therapy; Th, therapy; ES, electrical stimulation

**Fig. 7:**
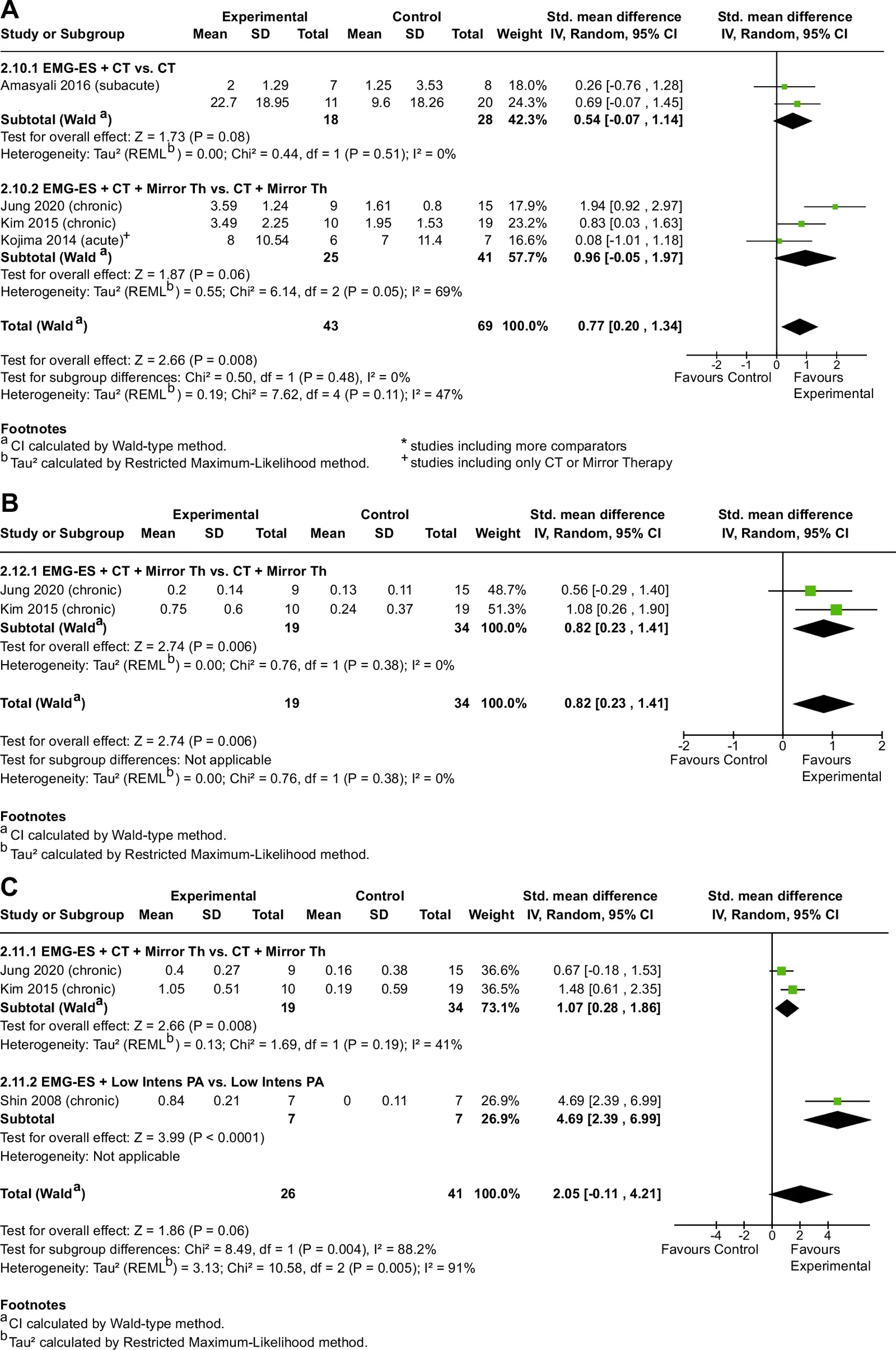
Meta-Analysis of the effects of EMG-ES combined with other interventions versus the comparators on the *body functions and structures* domain of the International Classification of Functioning, Disability and Health (ICF), assessed using grip strength (**A**), wrist flexion strength (**B**), and wrist extension strength (**C**). All were evaluated immediately after the intervention. Abbreviations: CT, conventional therapy; Th, therapy; PA, physical activity; ES, electrical stimulation

**Fig. 8:**
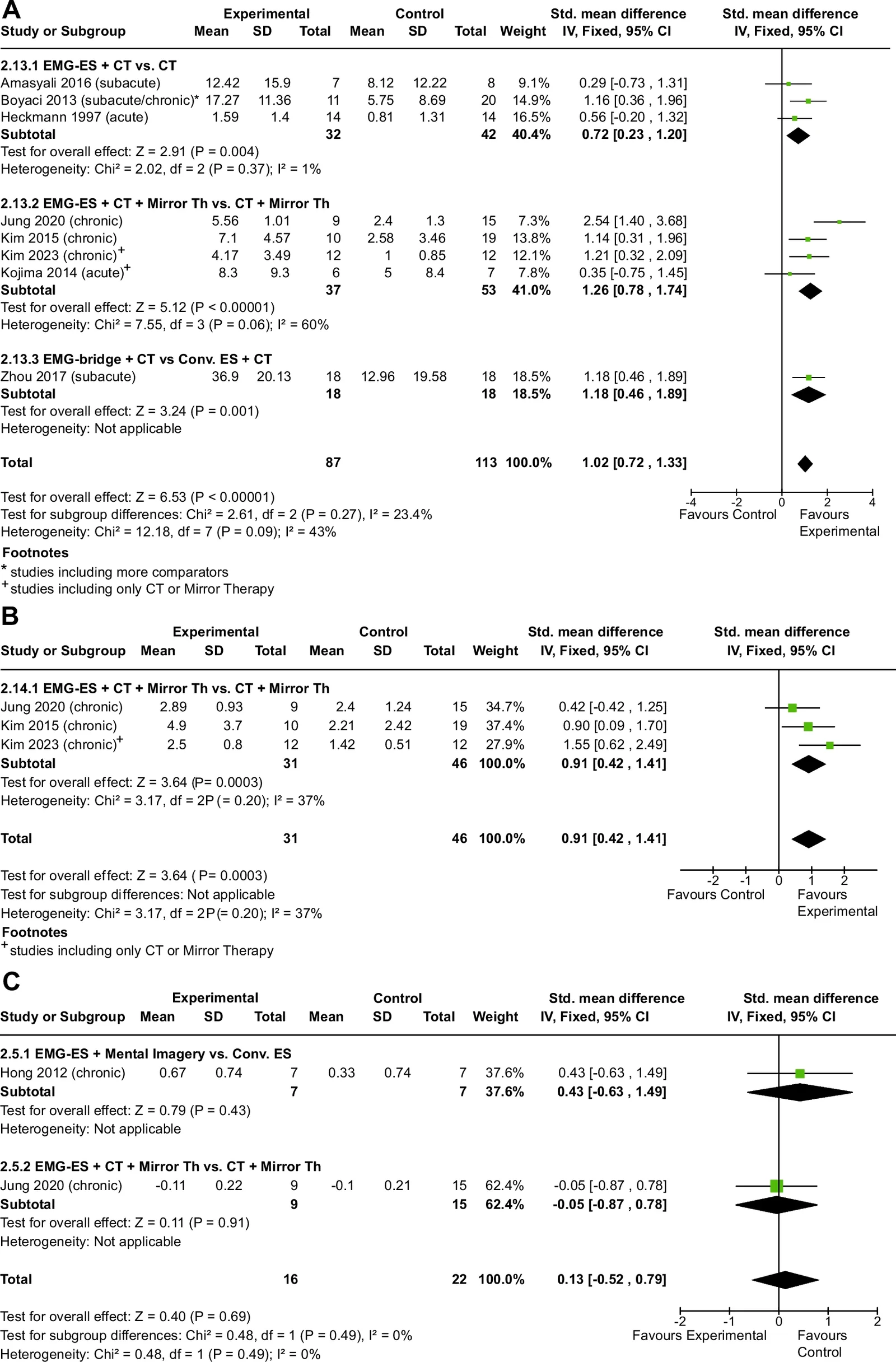
Meta-Analysis of the effects of EMG-ES combined with other interventions versus the comparators on the *body functions and structures* domain of the International Classification of Functioning, Disability and Health (ICF), assessed using wrist extension Range of Motion (ROM) (**A**), wrist flexion ROM (**B**), and Modified Ashworth Scale (MAS) (**C**). All were evaluated immediately after the intervention. Abbreviations: CT, conventional therapy; Th, therapy; PA, physical activity; ES, electrical stimulation

**Fig. 9:**
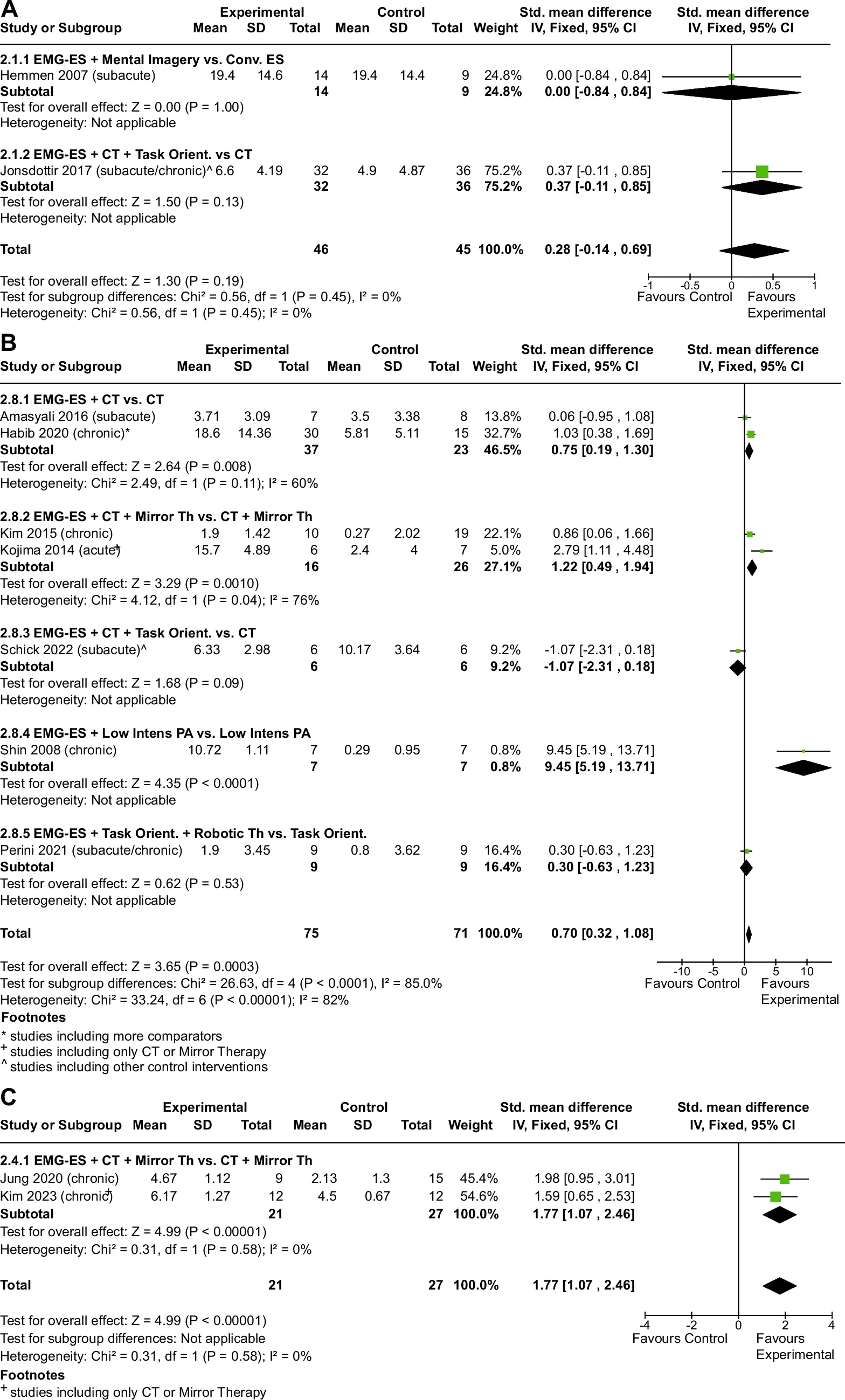
Meta-Analysis of the effects of EMG-ES combined with other interventions versus the comparators on the *activity* domain of the International Classification of Functioning, Disability and Health (ICF), assessed using Action Research Arm Test (ARAT) (**A**), Box and Blocks Test (BBT) (**B**), and Manual Function Test (MFT) (**C**). All were evaluated immediately after the intervention. Abbreviations: CT, conventional therapy; Th, therapy; ES, electrical stimulation

**Fig. 10:**
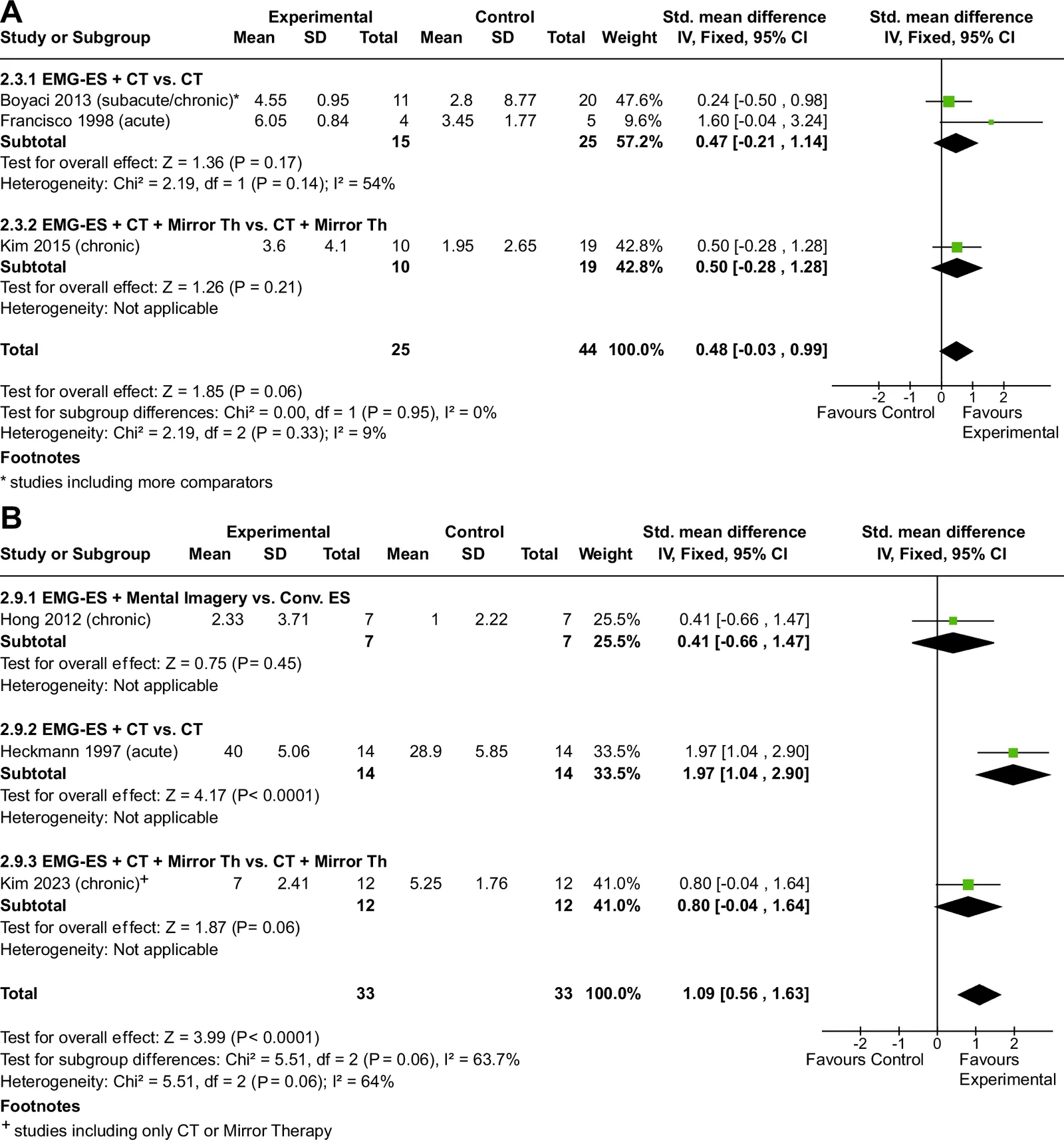
Meta-Analysis of the effects of EMG-ES combined with other interventions versus comparators on the *participation* domain of the International Classification of Functioning, Disability and Health (ICF), assessed using Functional Independence Measure (FIM) (**A**), and Barthel index (**B**). Both were evaluated immediately after the intervention. Abbreviations: CT, conventional therapy; Th, therapy; ES, electrical stimulation

**Fig. 11:**
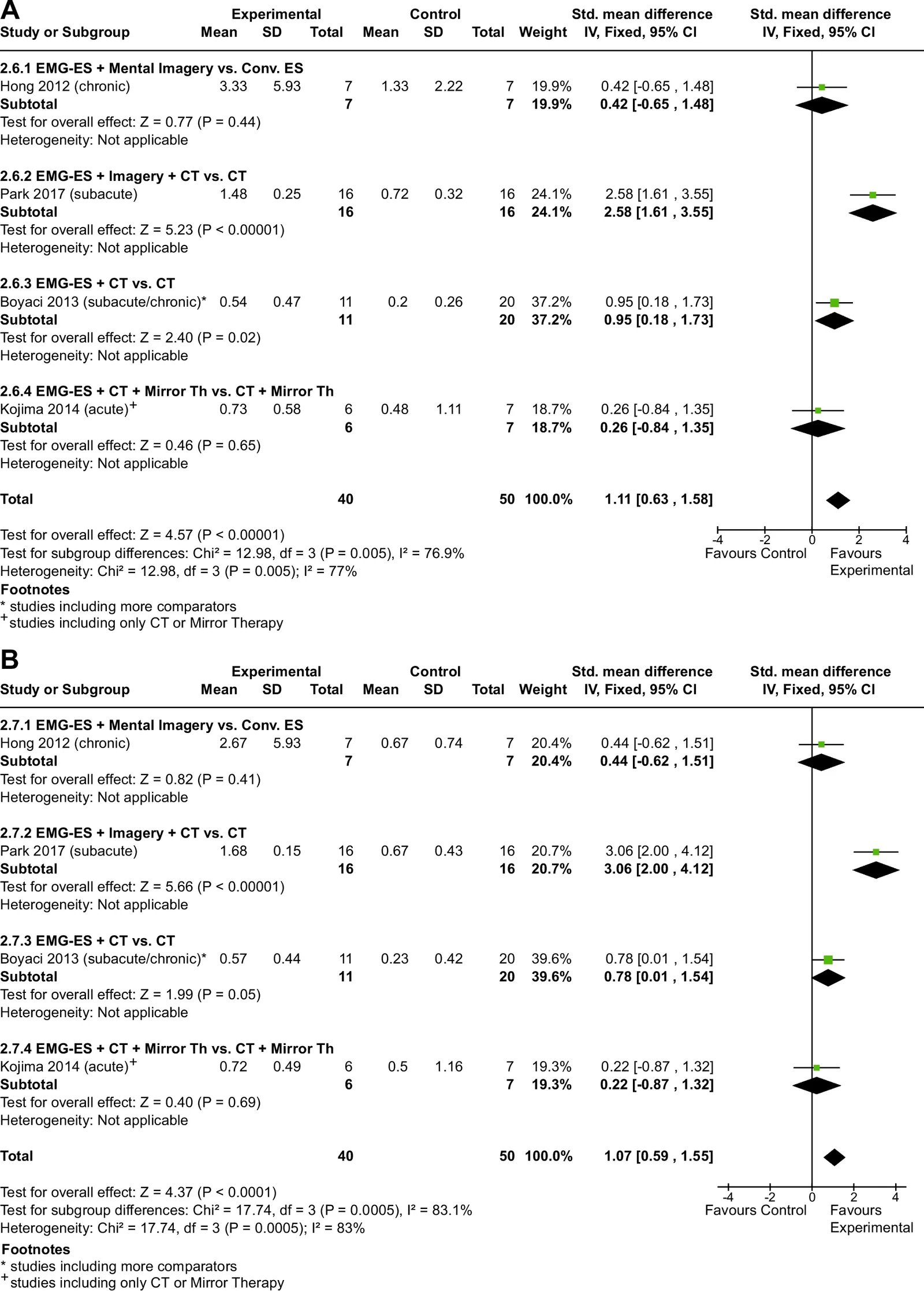
Meta-analysis of the effects of EMG-ES combined with other interventions versus comparators on the *patient-reported outcomes* domain of the International Classification of Functioning, Disability and Health (ICF), assessed using Motor Activity Log – Amount of Use (MAL-AOU) (**A**), and Motor Activity Log – Quality of Movement (MAL-QOM) (**B**). Both were evaluated immediately after the intervention. Abbreviations: CT, conventional therapy; Th, therapy; ES, electrical stimulation

Figures 6, 7, 8, 9, 10, and 11 present meta-analyses evaluating the effects of EMG-ES combined with other interventions relative to their comparators. In this category of interventions, the meta-analyses were stratified according to the type of combined treatment applied to the experimental group, in which EMG-ES was complemented by, for example, conventional therapy, mirror therapy and motor imagery, among others. A total of 14 meta-analyses are presented, each corresponding to a different clinical outcome and representing effects immediately after the intervention. Additional forest plots for the subsections of the FMA-UE and MAS are presented in **Supplementary Material **8.

##### Body functions and structures

Low certainty evidence indicated greater improvements in FMA-UE when EMG-ES was combined with other interventions (large SMD = 1.53, 95% CI = 1.24 to 1.81, Fig. 6). Very low certainty evidence indicated no differences between interventions in FMA-wrist (SMD = −0.01, 95% CI = −0.77 to 0.75, **Supplementary Material 8** Fig. 1), FMA-hand (SMD = 0.12, 95% CI = −0.65 to 0.89, **Supplementary Material 8** Fig. 2), and FMA-coordination/speed (SMD = 0.14, 95% CI = −0.62 to 0.90, **Supplementary Material 8** Fig. 3). Very low certainty evidence indicated greater improvements in grip strength (moderate SMD = 0.77, 95% CI = 0.20 to 1.34, Fig. 7A) and wrist flexion strength (moderate SMD = 0.82, 95% CI = 0.23 to 1.41, Fig. 7B) when EMG-ES was combined with other interventions. Very low certainty evidence indicated no difference between interventions in wrist extension strength (SMD = 2.05, 95% CI = −0.11 to 4.21, Fig. 7C). Very low certainty evidence indicated greater improvements in wrist extension ROM (moderate SMD = 1.02, 95%CI = 0.72 to 1.33, Fig. 8A) and wrist flexion ROM (moderate SMD = 0.91, 95% CI = 0.42 to 1.41, Fig. 8B) when EMG-ES was combined with other interventions. Low certainty evidence indicated no difference between interventions in MAS (SMD = 0.13, 95%CI = −0.52 to 0.79, Fig. 8C) and MAS-wrist flexor (SMD = −0.29, 95%CI = −0.82 to 0.25, **Supplementary Material 8** Fig. 4).

##### Activity

Low certainty evidence indicated no difference between interventions in ARAT (SMD = 0.28, 95% CI = −0.14 to 0.69, Fig. 9A). Very low certainty evidence indicated greater improvements in BBT (moderate SMD = 0.70, 95% CI = 0.32 to 1.08, Fig. 9B) and MFT (large SMD = 1.77, 95% CI = 1.07 to 2.46, Fig. 9C) when EMG-ES was combined with other interventions. A sensitivity analysis was performed for the meta-analysis evaluating BBT data, in which the results of the study by Shin et al. [43] were excluded because they diverged from the other studies and were deemed high risk. Excluding this study did not change the overall results of the meta-analysis (see **Supplementary Material 8** Fig. 5).

##### Participation

Very low certainty evidence indicated no difference between interventions in FIM (SMD = 0.48, 95% CI = −0.03 to 0.99, Fig. 10A), but greater improvements in Barthel index (moderate SMD = 1.09, 95% CI = 0.56 to 1.63, Fig. 10B) when EMG-ES was combined with other interventions.

##### Patient-Reported outcomes

Very low certainty evidence indicated greater improvements in MAL-AOU (moderate SMD = 1.11, 95% CI = 0.63 to 1.58, Fig. 11A) and MAL-QOM (moderate SMD = 1.07, 95% CI = 0.59 to 1.55, Fig. 11B) when EMG-ES was combined with other interventions.

#### Effects of EMG-based robotic platforms

Figures 6, 7, and 8 in **Supplementary Material ****8** present meta-analyses evaluating the effects of EMG-based robotic platforms compared with various control interventions. Given the heterogeneity of interventions, comparators varied considerably across studies; however, studies were pooled by intervention type (e.g., EMG-robotic therapy or EMG-robotic therapy + conventional therapy) to facilitate meta-analysis. A total of nine meta-analyses are presented, each corresponding to a different clinical outcome and/or time of assessment. Additional forest plots for the subsections of the FMA-UE and MAS are presented in **Supplementary Material **8.

##### Body functions and structures

Very low and low certainty evidence indicated no difference between interventions in FMA-UE immediately after (SMD = 0.02, 95% CI = −0.41 to 0.46, **Supplementary Material 8** Fig. 6A) and at follow up *≥ * 3 months (SMD = −0.29, 95% CI = −0.92 to 0.33, **Supplementary Material 8** Fig. 6B), respectively. Low to moderate certainty evidence indicated no difference between interventions in FMA-wrist (SMD = −0.38, 95% CI = −1.01 to 0.25, **Supplementary Material 8** Fig. 9), FMA-hand (SMD = −0.09, 95% CI = −0.59 to 0.41, **Supplementary Material 8** Fig. 10), and FMA-coor/speed (SMD = 0.34, 95% CI = −0.29 to 0.97, **Supplementary Material 8** Fig. 11) immediately after intervention, as well as no difference between interventions in FMA-hand at follow-up < 3 months (SMD = 0.13, 95% CI = −0.49 to 0.75, **Supplementary Material 8** Fig. 12). Low and very low certainty evidence indicated greater improvements for the EMG-based robotic platform in FMA-wrist/hand immediately after intervention (large SMD = 2.21, 95% CI = 1.45 to 2.97, **Supplementary Material 8** Fig. 13) and at follow-up *≥ * 3 months (large SMD = 1.53, 95% CI = 0.83 to 2.23, **Supplementary Material 8** Fig. 14), respectively. Very low certainty evidence indicated greater improvements for the EMG-based robotic platform in MAS-finger (large SMD = 1.74, 95% CI = −2.53 to −0.95, **Supplementary Material 8** Fig. 15) and MAS-wrist (moderate SMD = −1.03, 95% CI = −1.61 to −0.45, **Supplementary Material 8** Fig. 16) immediately after intervention. Low certainty evidence indicated greater improvements for EMG-based robotic platform in MAS-wrist at follow-up *≥ * 3 months (large SMD = −2.32, 95% CI = −3.20 to −1.45, **Supplementary Material 8** Fig. 17). Very low certainty evidence indicated greater improvements for the EMG-based robotic platform in WMFT-Time immediately after intervention (moderate SMD = −0.82, 95% CI = −1.43 to −0.21, **Supplementary Material 8** Fig. 6C).

##### Activity

Low and very low certainty evidence indicated no difference between interventions in ARAT immediately after intervention (SMD = 0.08, 95% CI = −0.29 to 0.45, **Supplementary Material 8** Fig. 7A) and at follow-up *≥ * 3 months (SMD = 0.52, 95% CI = −0.05 to 1.09, **Supplementary Material 8** Fig. 7B), respectively.

##### Participation

Very low certainty evidence indicated no difference between interventions in FIM immediately after intervention (SMD = −0.38, 95% CI = −0.95 to 0.18, **Supplementary Material 8** Fig. 8A) and at follow-up *≥ * 3 months (SMD = −0.33, 95% CI = −0.89 to 0.23, **Supplementary Material 8** Fig. 8B).

##### Patient-Reported outcomes

Very low and low certainty evidence indicated no difference between interventions in MAL-AOU (SMD = −0.11, 95% CI = −0.61 to 0.38, **Supplementary Material 8** Fig. 8C) and MAL-QOM (SMD = 0.10, 95% CI = −0.50 to 0.69, **Supplementary Material 8** Fig. 8D), respectively, immediately after intervention.

#### Effects of EMG biofeedback combined with other interventions

Figure 18 in **Supplementary Material ****8** presents the meta-analyses for evaluating the effects of EMG biofeedback combined with other interventions. Due to the limited number of studies identified and included in this subsection, only two meta-analyses could be conducted, both analyzing data collected immediately after the intervention. The meta-analyses include subgroups stratified by the additional intervention used in combination with EMG biofeedback.

##### Body functions and structures

Very low and low certainty evidence indicated greater improvements in wrist extension ROM (large SMD = 1.42, 95% CI = 0.99 to 1.86, **Supplementary Material 8** Fig. 18A) and Brunnstrom score (moderate SMD = 0.62, 95% CI = 0.13 to 1.12, **Supplementary Material 8** Fig. 18B), respectively, when EMG biofeedback combined with other interventions.

### Evidence gap map

**Fig. 12:**
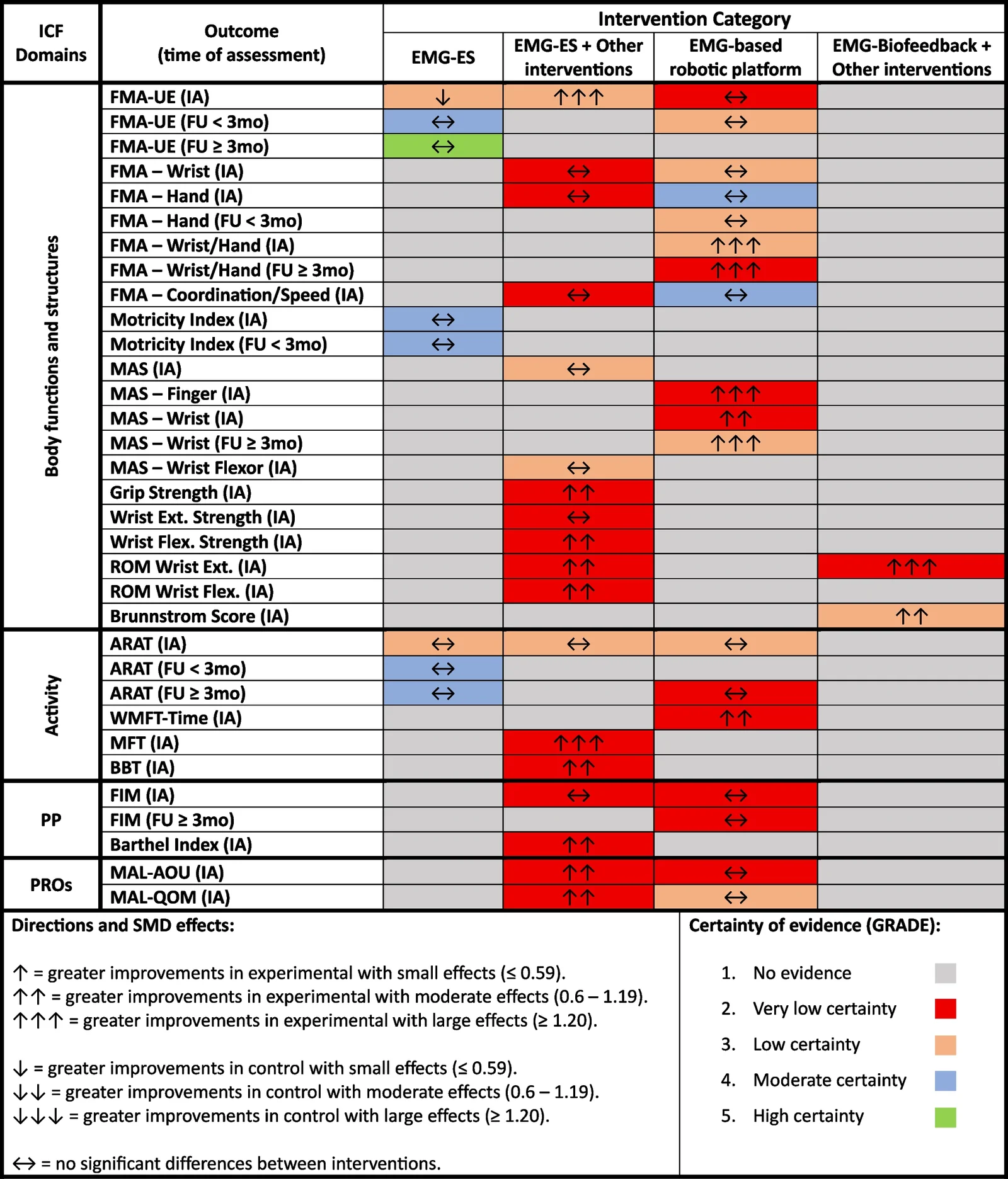
Evidence gap map of the included studies, segmented by each category of EMG-based intervention. Clinical outcomes with sufficient data for meta-analysis were organized according to the domains of the International Classification of Functioning, Disability and Health (ICF). Abbreviations: IA, immediately after; FU, follow-up. PP, Participation, PROs, Patient-reported outcomes. The evidence gap map is organized with ICF domains in rows and intervention categories in columns. Each cell summarizes the evidence for a specific intervention–outcome combination. Symbols indicate the direction and magnitude of effects (arrows show benefit direction and their number reflects effect size), while colors represent the certainty of evidence according to GRADE. Blank cells indicate combinations for which no evidence is currently available. This visualization facilitates the identification of both evidence-rich areas and remaining research gaps

The overall results, including the conclusions of the meta-analyses, the certainty of evidence, and the SMD magnitudes (effect directions), are presented in the evidence gap map (Fig. 12). This enables the identification of gaps by intervention type and clinical outcome, for which this systematic review cannot conclude due to insufficient data in the literature. It also highlights where the outcomes reported in the literature require higher-quality, better-structured studies to strengthen the evidence.

The evidence gap map revealed that the greatest concentration of evidence was found in outcomes assessed immediately after the intervention, while medium- and long-term assessments were limited. Consistent positive effects, often with moderate-to-large effect sizes, were observed in outcomes related to the *body functions and structures* domain, particularly when EMG-based interventions were combined with other therapeutic modalities. A distinct pattern was also observed for EMG-based robotic platforms, which demonstrated reductions in wrist and finger spasticity and sustained benefits during long-term follow-up, despite showing limited effects on functional outcomes. In contrast, evidence supporting the use of EMG-based biofeedback remained limited. Regarding *activity*-level outcomes, including ARAT, FIM, and hand function assessments, evidence generally indicated neutral effects or findings supported by low-certainty evidence, regardless of intervention type. Importantly, substantial areas of the map either lacked evidence or were supported only by very low- or low-certainty evidence. These gaps highlight the limited attention given to specific hand and wrist outcomes in the current literature and reinforce the need for high-quality clinical trials incorporating standardized assessments and longer follow-up periods to better define the clinical value of EMG-based interfaces.

## Discussion

This systematic review and meta-analysis evaluated the impact of EMG-based interventions on hand/wrist motor rehabilitation following stroke, integrating evidence from 38 studies (31 included in the meta-analyses) involving 1,132 participants. Overall, the findings indicate that the effectiveness of EMG-based interventions depends greatly on how they are applied. While EMG-ES, as an isolated therapy, did not show superiority over conventional interventions, its combination with other rehabilitation modalities was associated with clinically relevant benefits across all domains of the ICF model [24]. Similarly, EMG biofeedback combined with other therapies and EMG-based robotic platform interventions showed improvements over conventional therapy, particularly in ROM, motor function, and clinical outcomes for spasticity.

Previous reviews, such as those by Monte-Silva et al. [11] and Huo et al. [12], reported beneficial effects of EMG-ES and robotic interventions on upper limb function. In contrast, another review [13] could not establish conclusive evidence of the effectiveness of EMG-based interventions compared to non-EMG-based approaches. The present review was designed to comprehensively address limitations of previous reviews by: (1) comparing four categories of EMG-based interventions; (2) including a larger body of evidence; (3) organizing the meta-analyses according to specific clinical outcomes; and (4) classifying clinical assessments within the ICF framework. Accordingly, although previous reviews acknowledged potential benefits of EMG-ES combined with other therapies, they were unable to determine which specific EMG-based interventions were most effective compared with control interventions [11, 13]. The present review addresses this gap by identifying intervention-specific effects and providing more targeted clinical recommendations for hand and wrist motor rehabilitation.

### EMG-Triggered electrical stimulation

The findings regarding EMG-ES showed that there are no additional benefits beyond those achieved with standard interventions [63, 64]. Specifically, the comparators in the meta-analyses included conventional therapy, conventional stimulation, and sham stimulation. These interventions represent appropriate comparators for randomized controlled trials [5, 63, 65]. For example, conventional therapy, which integrates multiple rehabilitation strategies such as stretching and fine and gross motor exercises, is considered the gold standard in motor rehabilitation [65]. Similarly, sham stimulation, which does not produce physiological effects, is regarded as the gold standard for determining intervention-specific effects [66].

Overall, our findings suggest that EMG-ES interfaces failed to demonstrate effects as an isolated intervention. One possible explanation is the lack of standardized intervention protocols. Key parameters, including EMG activation thresholds, therapy duration, and session frequency, varied considerably across studies. For example, the threshold used to detect EMG activity may influence the level of active patient participation during therapy [11], as higher thresholds could require greater voluntary muscle activation and potentially promote stronger motor unit recruitment. Similarly, differences in therapy duration and session frequency may affect the cumulative therapeutic dose, influencing clinical outcomes. This variability hinders direct comparison of results across different studies.

EMG-ES is a neuromuscular intervention in which recorded muscle activity is processed, and when it exceeds a predefined threshold, ES is delivered to the target muscle [5, 19]. Recording myoelectrical activity can guide interventions aimed at facilitating the control of neural mechanisms involved in motor tasks after stroke [67], which may increase motivation, commitment, participation, and promote human–machine interaction in rehabilitation [68]. Despite these benefits highlighted in the literature, interventions based exclusively on EMG-ES may not be optimal according to the recommended guidelines for hand rehabilitation after stroke [10], which is consistent with the findings of the present review. This is because the interventions focused on isolated muscle activation and did not integrate functional, task-oriented interventions or intensive, repetitive practice. Although EMG-ES alone is not recommended, combining it with other established interventions may improve clinical outcomes. This is particularly relevant because motor rehabilitation cannot rely on a single intervention due to marked intersubject variability, individual clinical characteristics, and differences in the type and severity of disability [10]. In this context, the implementation of combined interventions may be particularly beneficial, allowing different motor components to be addressed, including voluntary control, muscular coordination, sensorimotor integration, and task-oriented function [5, 19].

### EMG-Triggered electrical stimulation combined with other interventions

Large effects were observed in favor of EMG-ES combined with other interventions, such as conventional therapy, mirror therapy, task-oriented therapy, and motor imagery. These effects were mainly observed in the FMA and MFT tests, which quantify both the degree of motor impairment and the upper-limb functional capacity [69, 70]. On the one hand, the FMA is a gold-standard tool that assesses motor disability and recovery after stroke, including upper-limb function (including subscales), sensitivity, and stroke severity [69, 71]. The MFT can complement the FMA, as it measures performance in functional manual tasks related to activities of daily living [70]. Therefore, the effects observed in these assessments highlight the potential clinical value of combining EMG-ES with other interventions, which may contribute to improved motor recovery outcomes in stroke rehabilitation.

EMG-triggered ES combined with other interventions also showed improvements in other clinical assessments, such as the MAL, a self-reported outcome that assesses the use of the affected upper limb in activities of daily living after stroke [72]. This finding is consistent with the benefits observed in more objective clinical measures, such as the BBT, Barthel Index, muscle strength parameters, and ROM. In summary, the BBT is a standardized functional physical test to assess gross manual dexterity of the upper limb by the number of blocks transferred in 60 s [73]. The Barthel Index is a scale used to assess independence in basic activities of daily living [71], and strength and ROM are recommended clinical variables for assessing rehabilitation progress and detecting specific functional limitations [10]. Overall, the combination of interventions not only improves specific neuromuscular properties but also promotes functional transfer to activities of daily living, covering multiple ICF domains. Therefore, the impact of EMG-based interventions is maximized when integrated with other therapeutic modalities. For example, EMG-ES can facilitate voluntary activation by improving participant involvement and effort, while others can direct that activation within functional movement patterns, increasing the likelihood of motor improvement [10].

No additional benefits were observed for spasticity, grip strength, FIM, or ARAT. However, these findings should be interpreted cautiously because only a limited number of studies contributed to these meta-analyses.

### EMG-Based robotic platform

Six studies investigating EMG-based control of robotic platforms were included in the review, with comparators varying considerably between them. Overall, the results indicate that these types of interventions promote specific benefits in the hand/wrist in FMA, spasticity, an WMFT-time tests, when specifically compared to task-oriented interventions [35], passive movements including and not including robots [55, 61], and conventional therapy [7, 34, 56]. Robotic platforms that combine information about the patient’s motor intention, estimated from EMG signals, can provide slow and controlled movement assistance. This approach encourages active participant engagement during motor intention and execution, reinforcing sensorimotor coupling and neural plasticity mechanisms [74]. Different strategies can be adopted, such as gesture classification or continuous movement estimation [20]. Notably, continuous estimation enables more natural movement execution by improving neuromuscular control, rather than discrete classification-based control [9]. Consequently, robotic assistance during rehabilitation can facilitate motor function in the affected limb, thereby reducing spasticity and increasing motor capacity. In fact, these benefits were observed in this review, supporting the use of EMG-based robotic platforms as effective interventions for stroke rehabilitation.

For outcomes within the ICF domains of *body functions and structure* and *activity*—specifically the MAL (in both subscales), FIM, and ARAT—no additional improvements were observed. Nevertheless, robotic systems may present practical limitations in clinical settings because they often require technical assistance and increase intervention costs [75].

### EMG biofeedback combined with other interventions

Few studies have investigated the effects of EMG-based biofeedback on motor rehabilitation of the hand and wrist after stroke. In this review, only two meta-analyses were performed because only three eligible studies were available. Overall, the results indicated that interventions using EMG biofeedback combined with other therapies improve ROM in wrist extension and Brunnstrom scale outcomes. These findings contrast with a previous review that reported no additional benefits from combining interventions [76]. However, that review evaluated multiple joints across both the upper and lower limbs, whereas the present review focused exclusively on hand and wrist rehabilitation.

Biofeedback provides real-time information on electrical and physiological responses to auditory or visual stimuli for the participant, allowing them to adjust their responses to use preserved pathways after injury, relearn motor skills, and interact with devices [76]. A narrative review has highlighted EMG biofeedback’s performance in stroke rehabilitation [21], and other reviews have demonstrated its great potential for post-stroke dysphagia rehabilitation [77]. However, according to our review, knowledge regarding its use in hand/wrist rehabilitation is limited or has been little explored. In this context, EMG biofeedback has several applications in stroke rehabilitation, including serious games [78], exoskeleton control [79], and neurofeedback to promote motor learning [80]. None of the included studies used these alternatives; they maintained a classic biofeedback approach based on auditory or visual feedback. Therefore, an interesting alternative for rehabilitation interventions could be the incorporation of serious games or more physiological biofeedback.

Finally, this category was supported by low-certainty evidence and a limited number of studies, which may bias the observed effects. Therefore, further research is needed to determine whether these interfaces provide meaningful improvements in motor rehabilitation.

### Clinical and technical considerations for EMG-based interfaces

The effectiveness of EMG-based interventions needs to be interpreted in light of their clinical and technical feasibility, which can be substantially influenced by patient characteristics. Feasibility depends largely on the presence and quality of residual muscle activation, which may vary according to stroke severity, lesion characteristics, stage of recovery, and communication abilities [10]. For example, patients with severe motor impairment or flaccid musculature may generate little or no EMG activity in the target muscles [81], making it difficult to achieve reliable triggering or control of EMG-driven systems. In line with this, an EMG-ES intervention in patients with severe paresis and complete absence of voluntary finger extension in the first 14 days after stroke did not show clinically relevant benefits compared with other therapeutic approaches [19]. By contrast, in chronic stages, EMG activity may be detectable but can partly reflect compensatory or maladaptive activation patterns, rather than task-specific muscle recruitment [82]. A practical illustration is that an early, flaccid patient with no detectable EMG in the finger extensors is unlikely to benefit from an EMG-triggered interface, whereas a mildly impaired chronic patient with consistent but partly compensatory EMG bursts may still be a viable candidate for EMG-based triggering or biofeedback. Together, these examples suggest that EMG-based interfaces are unlikely to be equally suitable across all stroke severity and phases; instead, their clinical use should be guided by a careful assessment of residual EMG activity and the underlying movement strategy.

Technical aspects of EMG acquisition also play a critical role in determining whether EMG is a usable interface. Low signal-to-noise ratio, variable electrode–skin contact, and electrode shift across sessions can all degrade signal quality and reduce reliability. For instance, one study showed that small electrode displacements can significantly reduce the accuracy of hand gesture identification [83]. In addition, the limited spatial selectivity of standard bipolar EMG recordings makes them susceptible to crosstalk from adjacent muscles [84, 85], which reduces the specificity of the recorded activity and may lead to unintended triggering. These technical constraints interact with patient-related factors, such as altered motor unit recruitment [86], reduced voluntary activation [87], and impaired neuromuscular control [88], which can further limit the generation of consistent and reproducible EMG signals. In practical terms, EMG-triggered or EMG-controlled interventions are more likely to be viable in patients who can produce clear, repeatable EMG activation in the relevant muscles, and less suitable in those with absent, highly variable, or predominantly compensatory EMG patterns.

Beyond patient characteristics and signal quality, the practical implementation of EMG-based interfaces also deserves consideration. Most EMG-based interventions require skin preparation, electrode placement, signal calibration, and periodic adjustments during therapy sessions. These procedures increase therapist workload and treatment time and may limit adoption in routine clinical practice, particularly in busy or time-limited rehabilitation services. They may also reduce patient adherence to long-term rehabilitation programs and hinder the scalability of EMG-based technologies across different clinical settings. Therefore, the successful clinical implementation of EMG-based interfaces will depend not only on matching the appropriate interface to each patient’s needs but also on simplifying setup procedures and optimizing usability in real-world clinical environments.

### Limitations and strengths

There are some limitations that constrain the scope of this review. A primary limitation is the marked heterogeneity of intervention protocols, including differences in number of sessions, stimulation parameters, equipment, and intervention type. This heterogeneity was particularly evident in the categories “EMG-ES combined with other interventions” and “EMG-based robotic platforms”. Ideally, experimental and control groups would differ only by the presence or absence of an EMG-based component; however, in some studies included in the present review, additional interventions were provided exclusively to the experimental group alongside the EMG-based intervention. In these cases, it is difficult to determine whether between-group differences are attributable to the EMG-based interface, the added therapies, or their interaction. This situation was commonly observed in studies where EMG was not the primary focus but which nonetheless met our eligibility criteria. We attempted to mitigate the impact of this limitation on our conclusions by explicitly assessing the certainty of evidence and risk of bias for each outcome.

A second limitation, inherent to the current body of literature, is the generally low methodological quality of the studies included in the meta-analyses. The trials showed considerable variability in design and reporting, particularly regarding the randomization process and missing outcome data, both of which can introduce bias and exaggerate treatment effects. Overall, a substantial proportion of the included studies were judged to have a high risk of bias (44.7%) or to raise some concerns (34.2%), indicating important limitations in the evidence supporting EMG-based interventions for motor rehabilitation. This finding should be viewed as a warning for the field and underscores the urgent need for well-designed, adequately powered, multicenter randomized controlled trials with robust randomization and allocation concealment, complete outcome reporting, and standardized outcome measures. Multicenter recruitment is particularly important to enhance generalizability to diverse clinical populations [89] and to strengthen the overall evidence base for EMG-based rehabilitation.

With respect to the meta-analyses, the restricted number of eligible studies and the inability to obtain raw numerical data from some authors [30, 44, 47, 49, 57] limited the possibility of performing more comprehensive quantitative syntheses. In addition, given the objectives of this review and the available data, we were unable to conduct meta-analyses stratified by stroke stage (acute, subacute, or chronic), which limits the ability to determine at which stage specific interventions might be most effective. As a partial alternative, we reported the stroke stage of the populations included in each meta-analysis. Consequently, the results of this review can be considered across different stroke stages, but they should be interpreted cautiously in light of these constraints.

Finally, the categorization strategy adopted for EMG-based interventions was necessary to identify more precisely the contexts in which EMG use appears to be associated with beneficial or neutral effects. In parallel, classifying clinical assessments according to the ICF framework [24] and conducting separate meta-analyses for each assessment allowed us to highlight outcome-specific patterns of improvement. The inclusion of evidence gap maps further helped to visualize gaps in the current literature, facilitate direct comparisons between intervention types and outcomes, and delineate areas where the evidence remains insufficient and where future research is most needed.

### Further research

From a clinical and methodological perspective, future studies should prioritize treatment fidelity by providing detailed intervention protocols, standardized training procedures, and rigorous allocation concealment. Larger sample sizes and broader inclusion criteria are also needed; in particular, including both ischemic and hemorrhagic stroke, as previous evidence suggests minimal differences in functional gains between these stroke types during rehabilitation [65]. From a technological perspective, there is a clear opportunity to explore rehabilitation interfaces based on high-density EMG (HD EMG), which was not implemented in any of the studies included in this review. HD EMG offers higher spatial resolution and enables more detailed characterization of motor function, motor unit behavior, and neural drive [88, 90], potentially providing a more informative control signal or an advanced biofeedback modality. Given the overall low to very low certainty of the available evidence, future randomized trials should report not only clinical outcomes but also EMG feasibility metrics (e.g., proportion of participants with usable signals, frequency of detection failures) and signal quality indices, so that the clinical and technical boundaries of EMG-based interfaces in stroke rehabilitation can be more clearly defined.

Due to the extensiveness of this topic, many questions remain open and would also be addressed in future research. For example, can an HD EMG-based interface provide clinically meaningful advantages over conventional bipolar EMG? Can artificial intelligence-based methods extract physiologically meaningful variables from EMG signals that can serve as biomarkers for motor rehabilitation? Which specific combination of EMG-triggered ES with complementary rehabilitation approaches (e.g., task-oriented training, mirror therapy, robotic therapy, or motor imagery) maximizes functional recovery, and what mechanisms underlie these effects? For EMG-based robotic platforms, which EMG-derived control strategies are most effective for improving motor recovery when integrated into robotic rehabilitation platforms? For EMG-based biofeedback interventions, should feedback primarily emphasize activation amplitude, contraction duration, timing, or more complex muscle activation patterns? Finally, how can EMG-based interfaces be simplified to reduce setup time and therapist workload while maintaining their clinical effectiveness?

## Conclusion

This systematic review, which includes an evidence gap map and meta-analysis, suggests that EMG-based interfaces for hand and wrist motor rehabilitation after stroke may be more effective when combined with conventional interventions. While EMG-ES alone did not clearly demonstrate superior clinical benefits compared to conventional therapies, its combination with other therapy modalities was associated with improvements across four domains of the International Classification of Functioning, Disability and Health (ICF) model: body functions and structures, activity, participation, and patient-reported outcomes. However, the certainty of the evidence for this category was rated as very low to low, indicating that the true effects may differ significantly from the observed estimates. Additionally, EMG-based robotic platforms may show specific effectiveness in reducing finger and wrist spasticity, although they did not demonstrate clear additional benefits in overall functional activity. Similarly, EMG-based biofeedback interfaces were associated with improvements in joint mobility and motor control in individual trials, but their overall effectiveness remains uncertain due to the limited number of available studies and the low to very low certainty of the evidence. Overall, these findings suggest that EMG-based interfaces should currently be considered complementary tools within comprehensive post-stroke rehabilitation programs, rather than stand-alone replacements for conventional therapy. Higher-quality, adequately powered randomized trials are needed to strengthen and refine current recommendations.

## Supplementary Information

The study contains supplementary material. Eight files are available with the current study.

Supplementary file 1 (docx 23 KB)

Supplementary file 2 (docx 18 KB)

Supplementary file 3 (docx 18 KB)

Supplementary file 4 (docx 40 KB)

Supplementary file 5 (docx 16 KB)

Supplementary file 6 (docx 41 KB)

Supplementary file 7 (docx 61 KB)

Supplementary file 8 (docx 3326 KB)

## Data Availability

No datasets or codes were generated or analyzed during the current study.
