# Supplementary material for: Impact of EMG-based interfaces for hand motor rehabilitation in stroke: A systematic review with evidence gap map and meta-analysis
Source: Med Biol Eng Comput. 2026 Aug 28;64(10):3675–701. doi: 10.1007/s11517-026-03660-7 (PMC13723694; doi:10.1007/s11517-026-03660-7)
Supplement: Supplementary file 1 [file 11517_2026_3660_MOESM1_ESM.docx]

# Supplementary file 1. Search strategy

## General search strategy

**TOTAL**: 3739

("stroke rehabilitat*" OR stroke OR "post-stroke" OR hemiplegia OR hemip*) AND (EMG OR electromyogr* OR "high-density electromyography" OR HD-EMG OR "electromyography-driven therapy*" OR "HD electromyography") AND ("hand function recovery" OR "motor recovery" OR "motor rehabilitation" OR "functional recovery" OR rehabilitation*) AND ("conventional therapy" OR "standard therapy" OR "traditional therapy" OR "therap*" OR treatment*) AND (hand* OR wrist* OR forearm* OR "upper limb*" OR finger* OR "upper extremity*" OR "manual" OR "fine motor skills")

## Search strategy by database

1. **WOS:** Up to 17/11/2025

**Results** – 685

**Filters:** Articles, English

ALL=(("stroke rehabilitat*" OR stroke OR "post-stroke" OR hemiplegia OR hemip*) AND (EMG OR electromyogr* OR "high-density electromyography" OR HD-EMG OR "electromyography-driven therapy*" OR "HD electromyography") AND ("hand function recovery" OR "motor recovery" OR "motor rehabilitation" OR "functional recovery" OR rehabilitation*) AND ("conventional therapy" OR "standard therapy" OR "traditional therapy" OR "therap*" OR treatment*) AND (hand* OR wrist* OR forearm* OR "upper limb*" OR finger* OR "upper extremity*" OR "manual" OR "fine motor skills"))

2. **SCOPUS:** Up to 17/11/2025

**Results –** 957

**Filters:** English

TITLE-ABS-KEY ( ( "stroke rehabilitat*" OR stroke OR "post-stroke" OR hemiplegia OR hemip* ) AND ( EMG OR electromyogr* OR "high-density electromyography" OR HD-EMG OR "electromyography-driven therapy*" OR "HD electromyography" ) AND ( "hand function recovery" OR "motor recovery" OR "motor rehabilitation" OR "functional recovery" OR rehabilitation* ) AND ( "conventional therapy" OR "standard therapy" OR "traditional therapy" OR "therap*" OR treatment* ) AND ( hand* OR wrist* OR forearm* OR "upper limb*" OR finger* OR "upper extremity*" OR "manual" OR "fine motor skills" ) )

3. **PUBMED:** Up to 17/11/2025

**Results –** 730

**Filters:** English

("stroke rehabilitat*" OR stroke OR "post-stroke" OR hemiplegia OR hemip*) AND (EMG OR electromyogr* OR "high-density electromyography" OR HD-EMG OR "electromyography-driven therapy*" OR "HD electromyography") AND ("hand function recovery" OR "motor recovery" OR "motor rehabilitation" OR "functional recovery" OR rehabilitation*) AND ("conventional therapy" OR "standard therapy" OR "traditional therapy" OR "therap*" OR treatment*) AND (hand* OR wrist* OR forearm* OR "upper limb*" OR finger* OR "upper extremity*" OR "manual" OR "fine motor skills")

4. **EMBASE:** Up to 17/11/2025

**Results –** 1204

**Filters**: N/A

("stroke rehabilitat*" OR stroke OR "post-stroke" OR hemiplegia OR hemip*) AND (EMG OR electromyogr* OR "high-density electromyography" OR HD-EMG OR "electromyography-driven therapy*" OR "HD electromyography") AND ("hand function recovery" OR "motor recovery" OR "motor rehabilitation" OR "functional recovery" OR rehabilitation*) AND ("conventional therapy" OR "standard therapy" OR "traditional therapy" OR "therap*" OR treatment*) AND (hand* OR wrist* OR forearm* OR "upper limb*" OR finger* OR "upper extremity*" OR "manual" OR "fine motor skills")

5. **DARE:** Up to 17/11/2025

**Results –** 12

**Filters:** N/A

("stroke rehabilitat*" OR stroke OR "post-stroke" OR hemiplegia OR hemip*) AND (EMG OR electromyogr* OR "high-density electromyography" OR HD-EMG OR "electromyography-driven therapy*" OR "HD electromyography") AND ("hand function recovery" OR "motor recovery" OR "motor rehabilitation" OR "functional recovery" OR rehabilitation*) AND ("conventional therapy" OR "standard therapy" OR "traditional therapy" OR "therap*" OR treatment*) AND (hand* OR wrist* OR forearm* OR "upper limb*" OR finger* OR "upper extremity*" OR "manual" OR "fine motor skills")

6. **PsycINFO:** Up to 17/11/2025

**Results –** 151

**Filters:** N/A

(Any Field: "stroke rehabilitat*" OR Any Field: stroke OR Any Field: "post-stroke" OR Any Field: hemiplegia OR Any Field: hemip*) AND (Any Field: EMG OR Any Field: electromyogr* OR Any Field: "high-density electromyography" OR Any Field: HD-EMG OR Any Field: "electromyography-driven therapy*" OR Any Field: "HD electromyography") AND (Any Field: "hand function recovery" OR Any Field: "motor recovery" OR Any Field: "motor rehabilitation" OR Any Field: "functional recovery" OR Any Field: rehabilitation*) AND (Any Field: "conventional therapy" OR Any Field: "standard therapy" OR Any Field: "traditional therapy" OR Any Field: "therap*" OR Any Field: treatment*) AND (Any Field: hand* OR Any Field: wrist* OR Any Field: forearm* OR Any Field: "upper limb*" OR Any Field: finger* OR Any Field: "upper extremity*" OR Any Field: "manual" OR Any Field: "fine motor skills")

7. **REHABDATA:** Up to 17/11/2025

**Results –** 0

**Filters:** Include International Research, English

**with all of these words** = stroke AND EMG AND Hand AND Conventional Therapy

**with all of these words** = stroke rehabilitat* AND electromyogr* AND Hand AND standard therapy AND Finger

**with all of these words** = "post-stroke" AND high-density electromyography AND motor recovery AND Hand

**with all of these words** = stroke rehabilitat* AND EMG AND traditional therapy

**with at least one of these words** = Hand OR Wrist OR Finger

**with all of these words** = "electromyography-driven therapy*" AND rehabilitation* AND hand AND "therap*" AND stroke
