# Supplementary material for: Impact of EMG-based interfaces for hand motor rehabilitation in stroke: A systematic review with evidence gap map and meta-analysis
Source: Med Biol Eng Comput. 2026 Aug 28;64(10):3675–701. doi: 10.1007/s11517-026-03660-7 (PMC13723694; doi:10.1007/s11517-026-03660-7)
Supplement: Supplementary file 2 [file 11517_2026_3660_MOESM2_ESM.docx]

# Supplemental file 2. Outcome category

According to the International Classification of Functioning, Disability and Health (ICF), the clinical assessments reported by the studies included in the systematic review can be clearly classified into four main domains.

1. **Body Functions and Structures**

These measures evaluate neuromuscular, biomechanical, or sensory impairments.

**Motor control and neuromuscular function**

- Fugl-Meyer Assessment (FMA) for Upper Extremity
- Brunnstrom scores (Upper limb and hand)
- Motricity Index
- Motor Status Scale (MSS)
- Chedoke-McMaster Stroke Assessment (CMSA-Hand)
- Motor Assessment Scale (MAS – arm items)
- Wolf Motor Function Test (WMFT) – Functional Ability Scale (FAS)

**Muscle strength**

- Grip strength (kg, N, hand ratio)
- Pinch strength (palmar, lateral, tip)
- Manual Muscle Testing (MMT)
- Wrist flexion/extension muscle strength
- Peak muscle torque (N)

**Range of motion (ROM)**

- Active ROM
- Wrist flexion/extension ROM
- MCP joint extension ROM
- Hand aperture

**Spasticity and muscle tone**

- Modified Ashworth Scale (MAS)
- Ashworth Scale (AS)

**Sensory function**

- FMA sensibility subscale
- Erasmus MC revised Nottingham Sensory Assessment (EmNSA-UE)

2. **Activity**

These assessments measure the ability to perform functional tasks with the affected upper limb.

**Upper-limb and hand activity measures**

- Action Research Arm Test (ARAT)
  - Total score and subscales: Grasp, Grip, Pinch, Gross Movement
- Wolf Motor Function Test (WMFT – time)
- Box and Block Test (BBT)
- Nine Hole Peg Test (NHPT)
- Frenchay Arm Test (FAT)
- Arm Motor Ability Test (AMAT)
- Upper Extremity Function Test (UEFT)
- Manual Function Test (MFT)
- General Jebsen–Taylor Hand Function Test (JTHFT)
- Minnesota Manual Dexterity Test (MMDT)
- Reaching Performance Scale (RPS)
- Drinking-from-a-glass task performance scales

3. **Participation**

These outcomes reflect independence and performance in activities of daily living.

**Participation-focused measures**

- Functional Independence Measure (FIM)
- Barthel Index (BI)
- Modified Barthel Index (MBI)
- Korean version of Barthel Index

4. **Patient-Reported Outcomes (PROs) / Quality of Life**

These measures capture the patient’s perspective.

**PRO Measures**

- Motor Activity Log (MAL)
  - Amount of Use (AOU)
  - Quality of Movement (QOM)
- Stroke Impact Scale (SIS)
  - Strength
  - ADL/IADL
  - Hand Function
  - Mobility
  - Recovery
- Stroke-Specific Quality of Life Scale (SS-QOL)
- Disability of the Arm, Shoulder and Hand questionnaire (DASH)
- Individually Prioritized Problem Assessment (IPPA)

**References**

1. Langhorne, P., Bernhardt, J., & Kwakkel, G. (2011). Stroke rehabilitation. *The Lancet*, *377*(9778), 1693-1702.
2. WHO. International Classification of Functioning, Disability and Health (ICF). 2001. https://www.who.int/standards/classifications/international-classification-of-functioning-disability-and-health (accessed January 06, 2026).
