# Supplementary material for: Impact of EMG-based interfaces for hand motor rehabilitation in stroke: A systematic review with evidence gap map and meta-analysis
Source: Med Biol Eng Comput. 2026 Aug 28;64(10):3675–701. doi: 10.1007/s11517-026-03660-7 (PMC13723694; doi:10.1007/s11517-026-03660-7)
Supplement: Supplementary file 3 [file 11517_2026_3660_MOESM3_ESM.docx]

# Supplementary file 3. GRADE domains

- **Risk of bias (Limitations in the design or execution of randomized trials)**

Evidence was not downgraded when all studies showed low risk of bias, downgraded when all showed high risk, or when there were some concerns. In mixed cases, sensitivity analyses were used to assess downgrading based on differences in effect-size magnitude between low- and high-risk studies (SMD thresholds: small ≤ 0.59, moderate 0.60–1.19, large ≥ 1.20).

- **Imprecision**

Judged using predefined SMD thresholds, with downgrading based on the number of thresholds crossed by the CI (one-level downgrading for crossing one threshold and two-level downgrading for crossing two thresholds), excluding the null effect.

- **Inconsistency**

Assessed using the I² statistic. No downgrading was applied when I² < 50%. When I² ≥ 50%, downgrading was considered only if all the following were present: lack of overlap of CIs, clear differences in effect estimates, and discrepancies between fixed- and random-effects models.

- **Indirectness**

Evaluated based on differences in study comparators within each meta-analysis subgroup. The certainty of evidence was downgraded by one level when comparators differed within the same subgroup analysis.

- **Publication bias**

Commonly evaluated by visual inspection of funnel plot asymmetry, it was assessed when at least five studies were pooled. The evidence was downgraded by one level if asymmetry was observed in the funnel plot.

We upgraded the certainty of evidence by one level (effect size) when a large effect size (SMD ≥ 1.20) was observed, and its confidence interval did not overlap with effect estimates of smaller magnitude.
