# Supplementary material for: Impact of EMG-based interfaces for hand motor rehabilitation in stroke: A systematic review with evidence gap map and meta-analysis
Source: Med Biol Eng Comput. 2026 Aug 28;64(10):3675–701. doi: 10.1007/s11517-026-03660-7 (PMC13723694; doi:10.1007/s11517-026-03660-7)
Supplement: Supplementary file 4 [file 11517_2026_3660_MOESM4_ESM.docx]

# Supplementary file 4. Studies excluded (after full-text screening)

A list of the studies excluded after the full-text screening is presented as follows which its respective reason of exclusion.

| Study | Reason for exclusion |
| --- | --- |
| Rong W, Tong KY, Hu XL, Ho SK. Effects of electromyography-driven robot-aided hand training with neuromuscular electrical stimulation on hand control performance after chronic stroke. Disabil Rehabil Assist Technol. 2015;10(2):149-159. | No control group |
| Papathanasiou I, Filipovic SR, Whurr R, Jahanshahi M. Plasticity of motor cortex excitability induced by rehabilitation therapy for writing. Neurology. 2003;61(7):977-980. | Type of study (Brief Communication) |
| Sawaki L, Butler AJ, Leng X, Wassenaar PA, Mohammad YM, Blanton S, Wittenberg GF. Differential patterns of cortical reorganization following constraint-induced movement therapy during early and late period after stroke: a preliminary study. NeuroRehabilitation. 2014;35(3):415-426. | Non-EMG-based intervention |
| Barker RN, Brauer S, Carson R. Training-induced changes in the pattern of triceps to biceps activation during reaching tasks after chronic and severe stroke. Exp Brain Res. 2009;196(4):483-496. | No hand/finger muscle intervention |
| Brauer SG, Hayward KS, Carson RG, Cresswell AG, Barker RN. The efficacy of SMART Arm training early after stroke for stroke survivors with severe upper limb disability: a protocol for a randomised controlled trial. BMC Neurol. 2013;13:71. | Non-EMG-based intervention |
| Park JH. Effects of mental imagery training combined electromyogram-triggered neuromuscular electrical stimulation on upper limb function and activities of daily living in patients with chronic stroke: a randomized controlled trial. Disabil Rehabil. 2020;42(20):2876-2881. | Inappropriate control group |
| Sunderland A, Tuke A. Neuroplasticity, learning and recovery after stroke: a critical evaluation of constraint-induced therapy. Neuropsychol Rehabil. 2005;15(2):81-96. | Non-EMG-based intervention |
| Daliri SS, Forogh B, Emami Razavi SZ, Ahadi T, Madjlesi F, Ansari NN. A single blind, clinical trial to investigate the effects of a single session extracorporeal shock wave therapy on wrist flexor spasticity after stroke. NeuroRehabilitation. 2015;36(1):67-72. | Non-EMG-based intervention |
| Conrad MO, Scheidt RA, Schmit BD. Effects of wrist tendon vibration on targeted upper-arm movements in poststroke hemiparesis. Neurorehabil Neural Repair. 2011;25(1):61-70. | Non-EMG-based intervention |
| Sorinola IO, White CM, Rushton DN, Newham DJ. Electromyographic response to manual passive stretch of the hemiplegic wrist: accuracy, reliability, and correlation with clinical spasticity assessment and function. Neurorehabil Neural Repair. 2009;23(3):287-294. | Non-EMG-based intervention |
| Hara Y, Obayashi S, Tsujiuchi K, Muraoka Y. The effects of electromyography-controlled functional electrical stimulation on upper extremity function and cortical perfusion in stroke patients. Clin Neurophysiol. 2013;124(10):2008-2015. | No control group |
| Hu C, Ti CHE, Shi X, Yuan K, Leung TW, Tong RKY. Development and External Validation of a Motor Intention–Integrated Prediction Model for Upper Extremity Motor Recovery After Intention-Driven Robotic Hand Training for Chronic Stroke. Arch Phys Med Rehabil. 2025;106(2):206-215. | Type of study (wrong design) |
| Platz T, Kaiser K, Laborn T, Laborn M. Effects of Intensive Impairment-Oriented Arm Rehabilitation for Chronic Stroke Survivors: An Observational Cohort Study. J Clin Med. 2024;14(1):176. | Type of study (wrong design) |
| Hu C, Ti CHE, Yuan K, Chen C, Khan A, Shi X, Tong RKY. Effects of high-definition tDCS targeting individual motor hotspot with EMG-driven robotic hand training on upper extremity motor function: a pilot randomized controlled trial. J Neuroeng Rehabil. 2024;21:169. | Intervention using other stimulation sources |
| Li Z, Li X, Fu X, Zhou T, Wang P, Fang L, Wang H. Modified tai chi movement training based on sEMG and movement analysis on improving upper extremities motor function: a protocol for a clinical randomised controlled trial. BMJ Open. 2024;14(10):e087399. | Non-EMG-based intervention |
| Choi JB, Cho KI. Effects of virtual reality-based robot therapy combined with task-oriented therapy on upper limb function and cerebral cortex activation in patients with stroke. Medicine (Baltimore). 2024;103(27):e38723. | Non-EMG-based intervention |
| Kolbasi EN, Huseyinsinoglu BE, Ozdemir Z, Bayraktaroglu Z, Soysal A. Priming constraint-induced movement therapy with intermittent theta burst stimulation to enhance upper extremity recovery in patients with stroke: protocol for a randomized controlled study. Acta Neurol Belg. 2024;124(3):887-893. | Non-EMG-based intervention |
| Chabhadiya C, Lohar D, Khan J. An observational study correlating the Modified Tardieu Scale and Hmax/Mmax ratio to evaluate wrist flexor spasticity in chronic stroke patients. Int J Pharm Clin Res. 2024. | Type of study (wrong design) |
| Tay SS, Zhang F, Visperas CA, Koh XH, Lau B, Neo JRE. Robot-mediated impairment-oriented and task-specific training on upper limb post stroke: feasibility and preliminary effects on physical function and quality of life. Front Neurol. 2024;15:1415773. | Non-EMG-based intervention |
| Wang S, Yang K, Zhu D, Rao R. Improvement of upper limb function in post-stroke patients with motion feedback training-based combination therapy: a retrospective analysis of muscle activation and recovery dynamics. Am J Transl Res. 2024;16(12):7618. | Non-EMG-based intervention |
| Liu Y, Dong X, Huo H, Feng L, Tong D, Liu J, Wang D. Effects of programmed flexor–extensor alternating electrical acupoint stimulation on upper limb motor functional reconstruction after stroke: study protocol for a double-blind, randomized controlled trial. Trials. 2023;24:324. | Non-EMG-based intervention |
| Khantan M, Avery M, Aung PT, Zarin RM, Hammelef E, Shawki N, Napoli A. The NuroSleeve, a user-centered 3D printed hybrid orthosis for individuals with upper extremity impairment. J Neuroeng Rehabil. 2023;20:103. | Type of study (wrong design) |
| Cocco ES, Goffredo M, Thouant CL, Infarinato F, Ottaviani M, Romano P, Franceschini M. Kinematics during the BBT: a pilot study on subjects with Parkinson's disease and post-stroke. Gait Posture. 2023;105:S14-S15. | Type of study (Conference) |
| Arantes, A. P., Bressan, N., Borges, L. R., & McGibbon, C. A. (2023). Evaluation of a novel real-time adaptive assist-as-needed controller for robot-assisted upper extremity rehabilitation following stroke. *Plos one*, *18*(10), e0292627. | No control group |
| Arjundas D. Electromyography (EMG) biofeedback for the rehabilitation of stroke patients. Int J Stroke. 2023;18(3):440. | Type of study (Conference) |
| Baumgartner, D., Nast, I., Bauer, C., Scheermesser, M., Sommer, B., Haas, M., Kool, J., Bansi, J. Holoreach – reaching exercises performing through augmented reality therapy – a feasibility study. JSES Int. 2023;7(5):1297 | Type of study (Conference) |
| Pournajaf S, Morone G, Straudi S, Goffredo M, Leo MR, Calabrò RS, et al. Neurophysiological and clinical effects of upper limb robot-assisted rehabilitation on motor recovery in patients with subacute stroke: a multicenter randomized controlled trial study protocol. Brain Sci. 2023;13(4):700. | Non-EMG-based intervention |
| Chang, S., Kim, J., Zistatsis, J., Liu, Y., Erickson, J., Bunnell, A., Boone, D. Gamified exercises and surface electromyography for rehabilitation in individuals with upper limb impairment. Prosthet. Orthot. Int. 2023;47(0): 225 | Type of study (Conference) |
| Yu Z, Yang X, Qin F, Ma T, Zhang J, Leng X, Liu X. Effects of acupuncture synchronized rehabilitation therapy on upper limb motor and sensory function after stroke: a single-center randomized controlled trial protocol. Front Neurol. 2023;14:1162168. | Non-EMG-based intervention |
| Seo NJ, Kamper DG, Ramakrishnan V, Harvey JB, Finetto C, Schranz C, et al. Effect of novel training to normalize altered finger force direction post-stroke: study protocol for a double-blind randomized controlled trial. Trials. 2022;23:301. | Non-EMG-based intervention |
| Jayavelu, J., Sethi, J., Kohli, S., Matin, T. Effect on electrical stimulation of tongue on hand functions in stroke patients. Int. J. Stroke. 2022;17(3):96-97 | Type of study (Conference) |
| Seo NJ, Barry A, Ghassemi M, Triandafilou KM, Stoykov ME, Vidakovic L, et al. Use of an EMG-controlled game as a therapeutic tool to retrain hand muscle activation patterns following stroke: a pilot study. J Neurol Phys Ther. 2022;46(3):198-205. | No control group |
| Song X, van de Ven SS, Chen S, Kang P, Gao Q, Jia J, Shull PB. Proposal of a wearable multimodal sensing-based serious games approach for hand movement training after stroke. Front Physiol. 2022;13:811950. | Non-EMG-based intervention |
| Chen JC, Shaw FZ. Recent progress in physical therapy of upper-limb rehabilitation after stroke: emphasis on thermal intervention. J Cardiovasc Nurs. 2006;21(6):469-473. | Non-EMG-based intervention |
| Dang R, Wang H. Effect of somatosensory simulation training combined with EMG biofeedback on upper limb dysfunction in stroke patients. Am J Transl Res. 2023;15(3):1842. | Intervention using other stimulation sources |
| Nam C, Rong W, Li W, Xie Y, Hu X, Zheng Y. The effects of upper-limb training assisted with an electromyography-driven neuromuscular electrical stimulation robotic hand on chronic stroke. Front Neurol. 2017;8:679. | No control group |
| Shi XQ, Heung HL, Tang ZQ, Li Z, Tong KY. Effects of a soft robotic hand for hand rehabilitation in chronic stroke survivors. J Stroke Cerebrovasc Dis. 2021;30(7):105812. | No control group |
| Saraefi, H.A.J., Büyükturan, Ö.Ü.B., Büyükturan, Ö. Comparison of the Effects of Extracorporeal Shock Wave Therapy and Dry Needing on Spasticity in Poststroke Patients. NeuroQuantology. 2022;20(13):772-790 | Non-EMG-based intervention |
| Brunfeldt A, Lum P, Bregman B. Exoskeletons increase paretic limb use in stroke survivors during a bimanual virtual reality reaching task. J Clin Transl Sci. 2022;6(s1):55-56. | Type of study (Conference) |
| Major ZZ, Vaida C, Major KA, Tucan P, Brusturean E, Gherman B, Pisla D. Comparative assessment of robotic versus classical physical therapy using muscle strength and ranges of motion testing in neurological diseases. J Pers Med. 2021;11(10):953. | Non-EMG-based intervention |
| de Almeida Oliveira R, Vieira CdS, Fernandes LFR, Patrizzi L, de Oliveira SF, Sande de Souza AP. Mental practice and mirror therapy associated with conventional physical therapy training on the hemiparetic upper limb in poststroke rehabilitation: a preliminary study. Top Stroke Rehabil. 2014;21(6):484-494. | Non-EMG-based intervention |
| Shi XQ, Ti CHE, Lu HY, Hu CP, Xie DS, Yuan K, Tong RKY. Task-oriented training by a personalized electromyography-driven soft robotic hand in chronic stroke: a randomized controlled trial. Neurorehabil Neural Repair. 2024;38(8):595-606. | Inappropriate control group |
| Pan B, Huang Z, Jin T, Wu J, Zhang Z, Shen Y. Motor function assessment of upper limb in stroke patients. J Healthc Eng. 2021;2021:6621950. | No control group |
| Huang Y, Nam C, Li W, Rong W, Xie Y, Liu Y, Hu X. A comparison of the rehabilitation effectiveness of neuromuscular electrical stimulation robotic hand training and pure robotic hand training after stroke: a randomized controlled trial. Biomed Signal Process Control. 2020;56:101723. | Inappropriate control group |
| Lee AY, Park SA, Park HG, Son KC. Determining the effects of a horticultural therapy program for improving the upper limb function and balance ability of stroke patients. HortScience. 2018;53(1):110-119. | Non-EMG-based intervention |
| Lee JJ, Shin JH. Predicting clinically significant improvement after robot-assisted upper limb rehabilitation in subacute and chronic stroke. Front Neurol. 2021;12:668923. | Non-EMG-based intervention |
| Prados-Román E, Cabrera-Martos I, López-López L, Rodríguez-Torres J, Torres-Sánchez I, Ortiz-Rubio A, Valenza MC. Deficits underlying handgrip performance in mildly affected chronic stroke persons. Top Stroke Rehabil. 2021;28(3):190-197. | Non-EMG-based intervention |
| Pundik, S., McCabe, J., Skelly, M., Salameh, A., Chen, Z., Tatsuoka, C., Fatone, S. Myoelectric upper limb orthosis use in motor learning therapy in chronic stroke. Neurorehabil. Neural Repair. 2021;35(11):NP14-NP15 | Type of study (Conference) |
| Akçay, S., Karagözoğlu Coşkunsu, D., Erkan Oğul, Ö., Akyol, D.K., Öztürk, N., Krespi, Y. The effect of robotic rehabilitation for recovery of hand functions in patients with acute stroke: Pilot study. Gait Posture. 2020;81:6-7 | Type of study (Conference) |
| Lu Z, Briley A, Zhou P, Li S. Are there trigger points in the spastic muscles? Electromyographical evidence of dry needling effects on spastic finger flexors in chronic stroke. Front Neurol. 2020;11:78. | No control group |
| Huang Y, Lai WP, Qian Q, Hu X, Tam EW, Zheng Y. Translation of robot-assisted rehabilitation to clinical service: a comparison of the rehabilitation effectiveness of EMG-driven robot hand assisted upper limb training in practical clinical service and in clinical trial with laboratory configuration for chronic stroke. Biomed Eng Online. 2018;17:91. | No control group |
| Amin F, Waris A, Iqbal J, Gilani SO, Rehman MZU, Mushtaq S, Tamam N. Maximizing stroke recovery with advanced technologies: a comprehensive assessment of robot-assisted, EMG-controlled robotics, virtual reality, and mirror therapy interventions. Results Eng. 2024;21:101725. | Type of study (Review) |
| Tsuchiya M, Morita A, Hara Y. Effect of dual therapy with botulinum toxin A injection and electromyography-controlled functional electrical stimulation on active function in the spastic paretic hand. J Nippon Med Sch. 2016;83(1):15-23 | No control group |
| Norouzi-Gheidari N, Archambault PS, Monte-Silva K, Kairy D, Sveistrup H, Trivino M, Milot MH. Feasibility and preliminary efficacy of a combined virtual reality, robotics and electrical stimulation intervention in upper extremity stroke rehabilitation. J Neuroeng Rehabil. 2021;18:61. | No control group |
| Page, S., Wilson, R., Chae, J. A Randomized Controlled Trial Comparing EMG-Triggered, Cyclic, and Sensory Electrical Stimulation. Arch. Phys. Med. Rehabil. 2019;100(10):37 | Type of study (Conference) |
| Akçay, S., Karagözoğlu Coşkunsu, D., Akyol, D.K., Oğul, Ö.E., Öztürk, N., Krespi, Y. Does emg-driven robotic treatment have effect for the recovery of the hand 9 years after stroke?. Gait Posture. 2019;73:362-363. | Type of study (Conference) |
| Jian C, Deng L, Liu H, Yan T, Wang X, Song R. Modulating and restoring inter-muscular coordination in stroke patients using two-dimensional myoelectric computer interface: a cross-sectional and longitudinal study. J Neural Eng. 2021;18(3):036005. | No control group |
| Qian Q, Nam C, Guo Z, Huang Y, Hu X, Ng SC, Poon W. Distal versus proximal—an investigation on different supportive strategies by robots for upper limb rehabilitation after stroke: a randomized controlled trial. J Neuroeng Rehabil. 2019;16:64. | Inappropriate control group |
| Ambrosini E, Gasperini G, Zajc J, Immick N, Augsten A, Rossini M, Krakow K. A robotic system with EMG-triggered functional electrical stimulation for restoring arm functions in stroke survivors. Neurorehabil Neural Repair. 2021;35(4):334-345. | No hand/finger muscle intervention |
| Camona C, Wilkins KB, Drogos J, Sullivan JE, Dewald JP, Yao J. Improving hand function of severely impaired chronic hemiparetic stroke individuals using task-specific training with the ReIn-Hand system: a case series. Front Neurol. 2018;9:923. | No control group |
| Patel J, Qiu Q, Yarossi M, Merians A, Massood S, Tunik E, Fluet G. Exploring the impact of visual and movement based priming on a motor intervention in the acute phase post-stroke in persons with severe hemiparesis of the upper extremity. Disabil Rehabil. 2017;39(15):1515-1523. | Non-EMG-based intervention |
| Poenaru AM, Cioroianu G, Balseanu AT, Banicioiu SC, Rogoveanu OC. Intermittent physical recovery has similar benefits to continuous physical recovery in patients in the acute and early sub‑acute stages following a stroke. Exp Ther Med. 2023;25(6):281. | Non-EMG-based intervention |
| Stein J, Narendran K, McBean J, Krebs K, Hughes R. Electromyography-controlled exoskeletal upper-limb–powered orthosis for exercise training after stroke. Am J Phys Med Rehabil. 2007;86(4):255-261. | No control group |
| Pehlivan YS, Armagan O. Effect of electrical stimulation combined with electromyographic biofeedback and exercise practices on upper extremity rehabilitation after stroke. Turk J Phys Med Rehabil. 2011;57(2):66-73. | Language |
| Chuang LL, Chen YL, Chen CC, Li YC, Wong AMK, Hsu AL, Chang YJ. Effect of EMG-triggered neuromuscular electrical stimulation with bilateral arm training on hemiplegic shoulder pain and arm function after stroke: a randomized controlled trial. J Neuroeng Rehabil. 2017;14:122. | No hand/finger muscle intervention |
| Turolla, A., Baldan, F., Baba, A., Mahmoud, A., Jakob, I. Effect of rehabilitation robotics controlled by semg closed-loop system, for the recovery of hand function after stroke. Neurorehabil. Neural Repair. 2018;32(4):324 | Type of study (Conference) |
| Doshi, D.B., Senthil Kumaran, D., Manikandan, N. Effect of observation with intent to imitate therapy on upper limb muscle activation in patients with stroke-randomized controlled trial. Neurorehabil. Neural Repair. 2018;32(4):400 | Type of study (Conference) |
| Turolla, A., Baldan, F., Baba, A., Mahmoud, A., Jakob, I. Rehabilitation robotics of hand function, after stroke: Diagnostic criteria for reference to therapy. Neurorehabil. Neural Repair. 2018;32(4):326 | Type of study (Conference) |
| Huber J, Kaczmarek K, Leszczyńska K, Daroszewski P. Post-stroke treatment with neuromuscular functional electrostimulation of antagonistic muscles and kinesiotherapy evaluated with electromyography and clinical studies in a two-month follow-up. Int J Environ Res Public Health. 2022;19(2):964. | Non-EMG-based intervention |
| Israely S, Carmeli E. Handwriting performance versus arm forward reach and grasp abilities among post-stroke patients, a case-control study. Top Stroke Rehabil. 2017;24(1):5-11. | Non-EMG-based intervention |
| Schick T, Schlake HP, Kallusky J, Hohlfeld G, Steinmetz M, Tripp F, Dohle C. Synergy effects of combined multichannel EMG-triggered electrical stimulation and mirror therapy in subacute stroke patients with severe or very severe arm/hand paresis. Restor Neurol Neurosci. 2017;35(3):319-332. | No control group |
| Uswatte, G., Taub, E., Bowman, M., Bryson, C., Morris, D., McKay, S., Barman, J., Mark, V. Persistent improvements in arm use in stroke survivors with plegic hands following expanded ci therapy. Arch. Phys. Med. Rehabil. 2017;98(10):11 | Type of study (Conference) |
| Griffin, C., Faieta, J., Persch, A., Peters, H., Page, S. Portable, EMG-triggered, myoelectric bracing: Restoring arm use and participation in stroke. Arch. Phys. Med. Rehabil. 2016;97(10):137-138. | Type of study (Conference) |
| Choi JB, Yang JE, Song BK. The effect of different types of resting hand splints on spasticity and hand function among patients with stroke. J Ecophysiol Occup Health. 2016;16(1/2):42. | Non-EMG-based intervention |
| Page S, Griffin C, White S. Efficacy of myoelectric bracing in moderately impaired stroke survivors: a randomized, controlled trial. J Rehabil Med. 2020;52(2):1-6. | No hand/finger muscle intervention |
| Uswatte G, Taub E, Bowman MH, Delgado A, Bryson C, Morris DM, Mark VW. Rehabilitation of stroke patients with plegic hands: randomized controlled trial of expanded Constraint-Induced Movement therapy. Restor Neurol Neurosci. 2018;36(2):225-244. | Non-EMG-based intervention |
| Qin, H., Wen, X., Bo, X. Effects of cross-education combined with electromyography biofeedback intervention on wrist dorsal extension function in patients with subacute stroke. Chinese Journal of Rehabilitation Medicine. 2024;39(9):1321-1326. | Language |
| Brown EVD, McCoy SW, Fechko AS, Price R, Gilbertson T, Moritz CT. Preliminary investigation of an electromyography-controlled video game as a home program for persons in the chronic phase of stroke recovery. Arch Phys Med Rehabil. 2014;95(8):1461-1469. | No control group |
| Krukowska J, Świątek E, Sienkiewicz M, Czernicki J. Influence of the surface electrostimulation controlled by muscle contraction on the bioelectric muscle activity and restoration of the hand function in cerebral stroke patients. NeuroRehabilitation. 2014;35(3):427-434. | Non-EMG-based intervention |
| Fardipour S, Bahramizadeh M, Arazpour M, Jafarpisheh AS, Azimian M. First prototype of EMG-controlled power hand orthosis for restoring hand extension in stroke patients. Proc Inst Mech Eng H. 2018;232(12):1176-1181. | No control group |
| Sedighipour, L., Rayegani, S.M., Anghouti, L., Rezazadeh, I., Tajziehchi, M., Bahrami, M.H., Raeis Sadat, S.A., Elyas Pour, D. The effect of neurofeedback and EMG-biofeedback therapy on improving hand function in stroke patients. Cerebrovasc. Dis. 2013;35:793. | Type of study (Conference) |
| Kagawa S, Koyama T, Hosomi M, Takebayashi T, Hanada K, Hashimoto F, Domen K. Effects of constraint-induced movement therapy on spasticity in patients with hemiparesis after stroke. J Stroke Cerebrovasc Dis. 2013;22(4):364-370. | Non-EMG-based intervention |
| Dorsch S, Ada L, Canning C. Is daily EMG-triggered electrical stimulation to multiple muscles of the affected upper limb a feasible intervention in very weak stroke? Int J Stroke. 2012;7:28. | Type of study (Conference) |
| Yurkewich A, Kozak IJ, Ivanovic A, Rossos D, Wang RH, Hebert D, Mihailidis A. Myoelectric untethered robotic glove enhances hand function and performance on daily living tasks after stroke. J Rehabil Assist Technol Eng. 2020;7. | No control group |
| Xia X, Dong X, Huo H, Zhang Y, Song J, Wang D. Clinical study of low-frequency acupoint electrical stimulation to improve thumb-to-finger movements after stroke: a randomized controlled trial. Medicine. 2023;102(47):e35755. | Non-EMG-based intervention |
| Basaran A, Emre U, Karadavut KI, Balbaloglu O, Bulmus N. Hand splinting for poststroke spasticity: a randomized controlled trial. Top Stroke Rehabil. 2012;19(4):329-337. | Non-EMG-based intervention |
| Inagaki Y, Seki K, Makino H, Matsuo Y, Miyamoto T, Ikoma K. Exploring hemodynamic responses using mirror visual feedback with electromyogram-triggered stimulation and functional near-infrared spectroscopy. Front Hum Neurosci. 2019;13:60. | No control group |
| Williams, S., Englefield, A., Singer, B., Mill, L. A repeated single case study pilot investigation combining EMG-FES and splinting to improve upper limb function in subacute stroke patients. Physiotherapy. 2011;97:eS1349-eS1350. | Type of study (Conference) |
| Inobe JI, Kato T. Effectiveness of finger-equipped electrode (FEE)-triggered electrical stimulation improving chronic stroke patients with severe hemiplegia. Brain Inj. 2013;27(1):114-119. | Non-EMG-based intervention |
| Lee, Y.H., Lee, J.H., Yoo, E.Y., Park, S.H., Choi, I.S., Kim, Y.H. EMG-triggered neuromuscular electrical stimulation combined with motor point block to improve on hand function in hemiplegic patients with severe spasticity. Artif. Organs. 2010;34(8):A35. | Type of study (Abstract) |
| Bello, A.I., Rockson, B.E., Olaogun, M.O. The effects of electromyographic-triggered neuromuscular electrical muscle stimulation on the functional hand recovery among stroke survivors. Afr J Med Med Sci. 2009;38(2):185-191. | Type of study (Abstract) |
| Hummelsheim H, Amberger S, Mauritz KH. The influence of EMG‐initiated electrical muscle stimulation on motor recovery of the centrally paretic hand. Eur J Neurol. 1996;3(3):245-254. | No control group |
| Prevo AJ, Visser SL, Vogelaar TW. Effect of EMG feedback on paretic muscles and abnormal co-contraction in the hemiplegic arm, compared with conventional physical therapy. Scand J Rehabil Med. 1982;14(3):121-131. | No randomization |
| Dziemian K, Kiper A, Baba A, Baldan F, Alhelou M, Agostini M, Kiper P. The effect of robot therapy assisted by surface EMG on hand recovery in post-stroke patients: a pilot study. Med Rehabil. 2018;21(4):4-10. | No control group |
| Hu XL, Tong RKY, Ho NS, Xue JJ, Rong W, Li LS. Wrist rehabilitation assisted by an electromyography-driven neuromuscular electrical stimulation robot after stroke. Neurorehabil Neural Repair. 2015;29(8):767-776. | No control group |
| Kojima, K., Ikuno, K., Tokuhisa, K., Shomoto, K. Clinical feasibility of mirror therapy combined with electromyography-triggered neuromuscular stimulation for the paretic hand after stroke: A pilot study. Rigakuryoho Kagaku. 2012;27(2):231-238. | Language |
| Araújo, R.C., Barbosa, M.P. Effects of conventional physical therapy and task specific training associated with electromyographic feedback to recover upper limb motor function after stroke. Motricidade. 2013;9(2):23-36. | Language |
| Kim GJ, Taub M, Creelman C, Cahalan C, O’Dell MW, Stein J. Feasibility of an electromyography-triggered hand robot for people after chronic stroke. Am J Occup Ther. 2019;73(4):7304345040p1-7304345040p9. | Type of study (Brief report) |
| Obayashi S, Takahashi R, Onuki M. Upper limb recovery in early acute phase stroke survivors by coupled EMG-triggered and cyclic neuromuscular electrical stimulation. NeuroRehabilitation. 2020;46(3):417-422. | No randomization |
| Kim SH, Park JH, Jung MY, Yoo EY. Effects of task‐oriented training as an added treatment to electromyogram‐triggered neuromuscular stimulation on upper extremity function in chronic stroke patients. Occup Ther Int. 2016;23(2):165-174. | Inappropriate control group |
| Cauraugh JH, Kim SB, Duley A. Coupled bilateral movements and active neuromuscular stimulation: intralimb transfer evidence during bimanual aiming. Neurosci Lett. 2005;382(1-2):39-44. | Inappropriate control group |
| Hesse S, Werner C, Pohl M, Rueckriem S, Mehrholz J, Lingnau ML. Computerized arm training improves the motor control of the severely affected arm after stroke: a single-blinded randomized trial in two centers. Stroke. 2005;36(9):1960-1966. | Non-EMG-based intervention |
| Marin-Pardo O, Donnelly MR, Phanord CS, Wong K, Pan J, Liew SL. Functional and neuromuscular changes induced via a low-cost, muscle-computer interface for telerehabilitation: a feasibility study in chronic stroke. Front Neuroergon. 2022;3:1046695. | No control group |
| Höhler C, Wild L, de Crignis A, Jahn K, Krewer C. Contralaterally EMG-triggered functional electrical stimulation during serious gaming for upper limb stroke rehabilitation: a feasibility study. Front Neurorobotics. 2023;17:1168322. | No clinical assessments |
| Cantillo-Negrete J, Rodríguez-García ME, Carrillo-Mora P, Arias-Carrión O, Ortega-Robles E, Galicia-Alvarado MA, Carino-Escobar RI. The ReHand-BCI trial: a randomized controlled trial of a brain-computer interface for upper extremity stroke neurorehabilitation. Front Neurosci. 2025;19:1579988. | Non-EMG-based intervention |
| Yu L, Xue PF, Qian LF, Feng W, Yao Q. Blood flow restriction training combined with muscle energy technology for upper limb functional recovery in poststroke spasticity: a randomised controlled trial protocol at a tertiary hospital in Jiaxing, China. BMJ Open. 2025;15(9):e092848. | Non-EMG-based intervention |
| Janmohamamdi S, Batouli SA, Ghabaee M, Yoonessi A, Hadjighassem M, Akbarfahimi N, Torkaman-Boutorabi A. Tractography-guided EMG Biofeedback in Stroke: an exploratory study of plasticity and motor outcomes. Arch Neurosci. 2025;12(12):e166080. | No control group |
| Liu Z, Wang L, Hou M, Li S, Zhang H, Li H. Combined therapy with contralateral controlled functional electrical stimulation and transcranial direct current stimulation for early post-stroke hand dysfunction. Biomed Eng Online. 2025;24:81. | Intervention using other stimulation sources |
| Xing Y, Zheng Y, Shang M. Effects of acupuncture-moxibustion combined with rehabilitation training on limb function and surface electromyography in patients with hemiplegia after stroke. J Acupunct Tuina Sci. 2025;1-9. | Non-EMG-based intervention |
| Spinazzola E, Mongardi A, Rossi F, Prestia A, Becchi S, Cavazzana R, Setti G. Motion tracking for automatically controlled functional electrical stimulation system in mirror therapy configuration: the enhanced Lazarus solution. IEEE Access. 2025. | Non-EMG-based intervention |
| Zhang Y, Zhao J, Dong J, Li S. Effectiveness of perception–interaction-enhanced rehabilitation in post-stroke recovery: a real-world propensity-matched cohort study. J Stroke Cerebrovasc Dis. 2025;108491. | No randomization |
| Wang L, Guo Z, Zhang J, Lin R, Tang X, Gao C, Xu N. Efficacy of acupuncture on upper limb dysfunction after stroke: a randomized controlled trial protocol with surface electromyography evaluation. Front Med. 2025;12:1615762. | Non-EMG-based intervention |
| Page SJ, Schaub K, Wilson RD, Chae J. Duration of EMG-triggered electrical stimulation treatment is not associated with upper extremity motor outcomes in subacute stroke. Disabil Rehabil. 2025;1-8. | No control group |
| Thorsen R, Ferrarin M. A wearable open-source neuroprosthesis/neuro-orthosis for restoring hand function. Sensors. 2025;25(11):3282. | No control group |
| Munoz-Novoa M, Andersson C, Sunnerhagen KS, Alt Murphy M. A novel intervention for upper limb rehabilitation in people with stroke combining myoelectric pattern recognition, virtual reality, and serious gaming: a qualitative study. Disabil Rehabil. 2025;47(15):3930-3937. | No control group |
| De Pasquale P, De Bartolo D, Russo M, Berger DJ, Maselli A, Borzelli D, d'Avella A. User-centered development of a personalized adaptive mirror therapy for upper-limb post-stroke rehabilitation using virtual reality and myoelectric control. Front Bioeng Biotechnol. 2025;13:1655416. | No control group |
| Qian F, Dai Y, Zheng G, Zhong Q, Lu J, Hu L, Chen Z. Bilateral versus unilateral training with rhythmic auditory cueing in stroke rehabilitation: effects on upper limb function and interhemispheric inhibition. Ann Clin Transl Neurol. 2025;12(11):2317-2327. | Non-EMG-based intervention |
| Ben Abdallah I, Bouteraa Y, Alotaibi A. AI-driven hybrid rehabilitation: synergizing robotics and electrical stimulation for upper-limb recovery after stroke. Front Bioeng Biotechnol. 2025;13:1619247. | No control group |
| Li H, Guo S, He R, Wang H, Kawanishi M. A home-based upper limb rehabilitation system via cloud-based teleoperation and sEMG-driven bilateral control. IEEE Internet Things J. 2025. | No control group |
| Cui Y, Cong F, Zeng M, Wang J. Effects and mechanisms of synchronous virtual reality action observation and electrical stimulation on upper extremity motor function and activities of daily living in patients with stroke: a protocol for a randomized controlled trial. Front Neurol. 2025;16:1499178. | Non-EMG-based intervention |
