# Supplementary material for: Impact of EMG-based interfaces for hand motor rehabilitation in stroke: A systematic review with evidence gap map and meta-analysis
Source: Med Biol Eng Comput. 2026 Aug 28;64(10):3675–701. doi: 10.1007/s11517-026-03660-7 (PMC13723694; doi:10.1007/s11517-026-03660-7)
Supplement: Supplementary file 5 [file 11517_2026_3660_MOESM5_ESM.docx]

# Supplementary file 5. Studies not included in meta-analysis

Summary of findings of the studies not included in the meta-analysis is presented as follows.

| Study | Main finding | Reason of exclusion |
| --- | --- | --- |
| Kirac-Unal et al. 2019 | This study showed that EMG-triggered electrical stimulation combined with task-oriented training produced superior motor and functional recovery of the paretic hand compared to conventional treatment. | Authors did not provide clinical scores |
| Cauraugh et al. 2002 | Both bilateral and unilateral training of the wrist/finger extensors in combination with EMG-triggered NMES improved motor capabilities, with bilateral protocol being superior. | Authors did not provide clinical scores |
| Kim et al. 2017 | Both conventional rehabilitation and conventional rehabilitation plus EMG biofeedback improved upper extremity function. | Authors did not provide clinical scores |
| Rayegani et al. 2014 | Both occupational therapy and occupational therapy plus EMG biofeedback improved hand function, without statistical differences between groups. | Outcomes not reported by other studies |
| Gabr et al. 2005 | Home-based electromyography-triggered  neuromuscular stimulation (ETMS) does not appear to convey a functional benefit to flaccid chronic stroke patients. However, ETMS consistently and markedly increases active wrist extension. | Authors did not provide clinical scores |
| Cauraugh et al. 2000 | EMG-triggered neuromuscular electrical stimulation to the wrist and finger extensors of individuals with chronic hemiparesis resulted in significant improvements for grasping small objects and for sustaining extensor contractions. | Authors did not provide clinical scores |
| Dorsch et al. 2014 | It is feasible to apply EMG-triggered electrical stimulation to multiple muscles of the upper limb in very weak people early after stroke. However, it does not appear to improve strength or activity beyond usual arm therapy that contains strengthening. | Outcomes not reported by other studies |
