# Supplementary material for: Impact of EMG-based interfaces for hand motor rehabilitation in stroke: A systematic review with evidence gap map and meta-analysis
Source: Med Biol Eng Comput. 2026 Aug 28;64(10):3675–701. doi: 10.1007/s11517-026-03660-7 (PMC13723694; doi:10.1007/s11517-026-03660-7)
Supplement: Supplementary file 6 [file 11517_2026_3660_MOESM6_ESM.docx]

# Supplementary file 6. Characteristics of the included studies

| Author (year) | N | Age (year) mean (STD) | Stage stroke | Protocol treatment | EMG-based Intervention | Control (non-EMG-based intervention) | Follow-up  Evaluation | Clinical outcome | Results |
| --- | --- | --- | --- | --- | --- | --- | --- | --- | --- |
| Hemmen, 2007 | EG: 14  CG: 9 | EG: 62.1 (12.7) CG: 60.7 (12.3) | Subacute | 60 sessions,  5/week for 12 weeks | EMG-triggered  feedback stimulation combined with movement imagery | Conventional electrostimulation | Yes (≥ 3mo) | ARAT, BFM | Movement imagery-assisted EMG-triggered feedback training was no more effective than conventional electrostimulation for improving arm–hand function or motor skills in stroke patients. |
| Unal, 2019 | EG: 12 CG: 11 | EG: 67 (50-79)*  CG: 64 (53-80)* | Acute/  Subacute | 20 sessions,  5/week for 4 weeks | EMG-triggered  Electrical stimulation combined with task-oriented training + conventional therapy | Conventional rehabilitation | Yes (≥ 3mo) | ARAT, FIM, Brunnstrom scores, Grip strength, SIS, | EMG-triggered electrical stimulation plus task-oriented training was more effective than conventional treatment for paretic hand recovery in acute/subacute stroke. |
| Thorsen, 2013 | EG: 5 CG: 6 | EG: 42.60(8.01)  CG: 56.50(8.77) | Subacute/  Chronic | 25 sessions,  3 to 5 /week for 5 to 8.3 weeks | Myoelectrically  controlled functional electrical stimulation | Sham stimulation | Yes (≥ 3mo) | ARAT, IPPA | Myoelectrically  controlled functional electrical stimulation produced clinically meaningful improvements in upper-limb function. |
| Kim, 2023 | EG: 12 CG: 12 | EG: 75.58(4.10)  CG: 74.91(4.27) | Chronic | 20 sessions,  5/week for 4 weeks | FES-based Mirror Therapy with Myoelectrical motion recognition + conventional Rehab | Conventional Mirror Therapy Intervention | No | FMA-UE, MFT, ROM (flexion, extension, pronation, supination), Korean BI, Brunnstrom scores | Combining FES-based mirror therapy with movement-recognition biofeedback resulted in significantly better upper-limb outcomes than conventional mirror therapy. |
| Perini, 2021 | EG: 9 CG: 9 | EG: 58.7(20.6) CG: 61.4(9) | Subacute/  Chronic | 20 sessions,  3 to 5/week for 3 to 5 weeks | Task-oriented therapy assisted with myoelectric signal-controlled functional electrical stimulation + Robotic Therapy | Conventional rehabilitation with task-oriented activities | No | FMA-UE, RPS, BBT | Task-oriented myoelectric signal-controlled functional electrical stimulation followed by robot therapy led to clinically meaningful upper-limb improvement in people with chronic hemiplegia. |
| Qian, 2017 | EG:14 CG: 10 | EG: 54.6(11.3)  CG: 64.6 (3.43) | Subacute | 20 sessions,  5/week for 4 weeks | Training assisted by the EMG-driven NMES-robotic arm system of repeated limb motions | Conventional rehabilitation | Yes (≥ 3mo) | FMA-UE, ARAT, FIM, MAS (wrist, finger) | NMES-robot-assisted upper-limb training produced intensive, clinically meaningful improvements that were maintained for 3 months and can complement traditional therapy. |
| Chen, 2022 | EG: 14 CG: 10 | EG: 54.58(10.98) CG:64.98(8.22) | Chronic | 12 sessions,  3/week for 4 weeks | EMG‑driven  robot‑assisted therapy (EMG biofeedback) | Task-oriented intervention | No | FMA-UE, WMFT (Time, FAS), MAL (AOU, QOM) | EMG-driven robot-assisted therapy matched task-oriented training in activity-level outcomes and improved task movement duration to a greater extent, but showed weaker effects in body function and participation-related measures. |
| Armagan, 2003 | EG: 14  CG: 13 | EG: 57(10.53) CG: 57.92(11.27) | Subacute | 20 sessions,  5/week for 4 weeks | Brunnstrom’s neurophysiologic approach + EMG biofeedback | Brunnstrom’s neurophysiologic approach + Placebo EMG biofeedback | No | ROM (wrist extension), Brunnstrom’s scores, | EMG biofeedback combined with neurophysiologic rehabilitation showed potential to improve hand function in hemiplegic patients. |
| Lourenção, 2008 | EG: 31 CG: 28 | EG: 53.71(12.19) CG: 57.11(13.65) | Chronic | 104 sessions,  2/week for 52 weeks | Occupational Therapy + Functional Electrical Stimulation + EMG-Biofeedback | Occupational Therapy + Functional Electrical Stimulation | No | Hand function test, MMDT, ROM (wrist), MAS (wrist) | The combination of EMG-Biofeedback, occupational therapy, and FES produced superior improvements at 6 and 12 months compared with conventional rehabilitation plus FES. |
| Coskunsu, 2022 | EG: 11 CG: 9 | EG: 59.9(14.2)  CG: 70(14) | Acute | 15 sessions,  5/week for 3 weeks | Conventional-therapy + EMG-driven robotic exoskeleton | Conventional therapy | No | FMA-UE, ARAT, MAL (AOU, QOM), Force | Robotic rehabilitation added to traditional therapy in the acute period provided no additional benefit. |
| Schick, 2022 | EG: 6 CG: 6 | EG: 68(7) CG: 62(9) | Subacute | 15 sessions, 5/week for 3 weeks | Task-oriented EMG-triggered multichannel functional electrical stimulation + Conventional rehabilitation | Single-channel cyclic neuromuscular electrical stimulation + Conventional rehabilitation | No | FMA-UE, BBT, SIS, | EMG-triggered multichannel functional electrical stimulation was superior to single-channel electrical stimulation in improving motor control and self-reported recovery measures. No statistical differences between groups were reported. |
| Jung, 2020 | EG:9 CG:15 | EG: 55.44(10.71)  CG1: 57.43(16.93)  CG2: 56.5(6.41) | Chronic | 60 sessions,  10/week for 6 weeks | Traditional physical therapy + Mirror therapy training combined with EMG-triggered FES | Mirror therapy OR physical therapy training (combination of groups) | No | ROM (wrist flexion and extension), Muscle strength, Grip strength, MAS, MFT | EMG-triggered FES combined with mirror therapy produced positive upper-limb recovery in chronic stroke patients, supporting its use as a promising rehabilitation program. |
| Aslan, 2012 | EG: 20  CG: 20 | EG: 57.90(13.32) CG: 60.75(12.81) | Acute/  Subacute/ Chronic | 15 sessions,  5/week for 3 weeks | Electromyographic biofeedback treatment + hemiplegia rehabilitation program | Hemiplegia rehabilitation program (conventional therapy) | No | Ashworth scale, Brunnstrom scores, UEFT, FMA, BI, ROM (wrist extension) | EMG biofeedback is an effective adjunct to conventional rehabilitation for reducing spasticity and enhancing motor and activity outcomes in post-stroke hemiplegic patients. |
| Zhou, 2017 | EG: 18 CG: 18 | EG: 50.9(13.8) CG: 56.9(10) | Subacute | 40 sessions,  10/week for 4 weeks | Electromyographic bridge + Standard therapy | Cyclic neuromuscular electrical stimulation + Standard therapy | No | Brunnstrom scores, FMA-UE, ROM (wrist and finger extension), MSS | EMG-Bridge training combined with standardized therapy was more effective than cyclic NMES for short-term recovery after subacute stroke. |
| de Kroon, 2008 | EG:11 CG: 10 | EG: 57.4(8) CG: 60.9 (10.9) | Chronic | 126 sessions,  21/week for 6 weeks | EMG-NMES | Cyclic NMES | Yes (< 3mo) | ARAT, Grip strength, FMA-UE, Motricity index | EMG-triggered electrical stimulation was not superior to cyclic stimulation for improving motor function after stroke. |
| Shin, 2008 | EG: 7 CG: 7 | EG: 61(7.5) CG: 54.1(3.9) | Chronic | 100 sessions,  10/week for 10 weeks | Low-intensity physical activities + EMG-NMES | Low-intensity physical activities | No | BBT, strength | EMG-stimulation seems to be better than control to improve hand motor functions. |
| Cauraugh, 2002 | EG: 20 CG: 5 | EG: 63.7 (NI) CG: 63.7 (NI) | Chronic | 4 sessions for 2 weeks | Unilateral and bilateral wrist/finger movement groups with EMG-triggered neuromuscular stimulation | Stretching + Voluntary contraction | No | BBT | Both bilateral and unilateral EMG-triggered NMES training enhanced motor capabilities, but the bilateral protocol was more effective. |
| Habib, 2020 | EG: 30 CG: 15 | EG1: 57.87(4.92) EG2: 57.33(4.11)  CG: 57.93 (4.63) | Chronic | 24 sessions,  3/week for 8 weeks | EMG‑triggered electrical stimulation + hand physiotherapy OR Alternating EMG stimulation + hand physiotherapy  (Combination of groups) | Hand physiotherapy + placebo NMES | No | BBT, Strength | Adding EMG-triggered or alternating EMG stimulation to a structured physical therapy program improved hand function and peak muscle torque in chronic stroke patients. |
| Park, 2017 | EG: 16 CG: 16 | EG: NI CG: NI | Subacute | 20 sessions,  5/week for 4 weeks | Mental practice combined with electromyogram-triggered electrical stimulation + conventional rehabilitation | Conventional rehabilitation | No | FMA-UE, MAL (AOU, QOM) | Mental practice combined with EMG-NMES was more effective than occupational and physical therapy alone for improving upper-extremity function after stroke. |
| Kim, 2017 | EG: 15  CG: 15 | EG: 56.93(NI) CG: 53.26 (NI) | Chronic | 12 to 20 sessions,  3 to 5/week for 4 weeks | Conventional rehabilitation + training using EMG biofeedback | Conventional rehabilitation | No | FMA-UE, MFT, FIM | Both conventional rehabilitation and rehabilitation with EMG biofeedback improved upper-extremity function. |
| Rayegani, 2014 | EG: 10 CG: 10 | EG: 53(9.8) CG: 54 (8.2) | Subacute/  Chronic | 10 sessions,  5/week for 2 weeks | Occupational therapy + EMG-Biofeedback | Occupational therapy | No | JHFT | Both occupational therapy and occupational therapy + EMG biofeedback improved hand function, that is, decreased their time during the JHFT. |
| Amasyali, 2016 | EG: 7 CG: 17 | EG: 51.29(12.25) CG1: 58.78(12.12) CG2: 65.38(9.07) | Subacute | 15 sessions,  5/week for 3 weeks | Conventional rehabilitation + EMG-triggered stimulation | Conventional rehabilitation | Yes (≥ 3mo) | ROM (wrist extension), Grip force, BBT, FMA-UE | Both groups improved hand function, but adding EMG-NMES to conventional rehabilitation produced greater gains in wrist extension range of motion. |
| Kojima, 2014 | EG: 6 CG: 7 | EG: 70.7(10.3) CG: 67.7(15.5) | Acute | 40 sessions,  10/week for 4 weeks | Combination of mirror therapy and electromyography-triggered neuromuscular stimulation + conventional physical and occupational therapy | conventional physical and occupational therapy | No | FMA-UE, ROM (wrist extension), Grip strength, BBT, WMFT (time, FAS), MAL (AOU, QOM) | Adding EMG-NMES +mirror therapy to conventional rehab significantly improved hand function. |
| Gabr, 2005 | EG: 8  CG: 4 | EG: 62.8(7.4)  CG: 58.8(8.3) | Chronic | 80 sessions,  10/week for 8 weeks | Home-based electromyography-triggered neuromuscular stimulation | Home exercise program | No | FMA, ARAT, ROM (wrist extension) | Although home-based electromyography-triggered neuromuscular stimulation did not improve functional outcomes, it significantly increased active wrist extension in chronic flaccid stroke patients. |
| Boyaci, 2013 | EG: 11 CG: 20 | EG: 56.1(6.8)  CG1: 64.4(9.5) CG2: 57.6(16.4) | Subacute/  Chronic | 15 sessions,  5/week for 3 weeks | EMG-triggered (active) neuromuscular electrical stimulation + neurophysiologic program | Passive NMES + neurophysiologic program OR  placebo stimulation + neurophysiologic program  (combination of groups) | No | FMA-UE, MAL (AOU, QOM), FIM, MAS (finger and wrist), ROM (wrist and finger extension), Grip strength | EMG-NMES was more effective than placebo NMES to improve hand function. |
| Zhao, 2022 | EG: 8 CG: 8 | EG: 52.75(17.16)  CG: 53.88(10.70) | Subacute | 40 sessions,  10/week for 4 weeks | Electromyography bridge | NMES | Yes (< 3mo) | ROM (wrist extension), FMA-UE, MMT, BI | Both EMG-bridge and NMES improved upper limb function, with no differences between groups. |
| Heckmann, 1997 | EG: 14 CG: 14 | EG: 50.1(14) CG: 54(11) | Acute | 20 sessions,  5/week for 4 weeks | Conventional rehabilitation + EMG-triggered electrical muscle stimulation | Conventional rehabilitation | No | Spasticity, ROM (hand extensor), BI | EMG-triggered electrical muscle stimulation was effective in improving range of motion of paretic limbs in early-phase stroke patients. |
| Hong, 2012 | EG: 7 CG: 7 | EG: 53.43 (7.39) CG: 51.29 (11.67) | Chronic | 40 sessions,  10/week for 4 weeks | Mental imagery training combined with EMG-triggered electrical stimulation | FES | No | FMA-UE, MAL (AOU, QOM), MAS, modified BI | Mental imagery combined with EMG-triggered electrical stimulation improved motor function of the paretic extremity in patients with chronic stroke compared to FES. |
| Francisco, 1998 | EG: 4 CG: 5 | EG: 60.3 (15.6) CG: 69.6()16.2 | Acute | Different according to the participant | EMG-triggered neuromuscular stimulation + Conventional rehabilitation | Conventional rehabilitation | No | FMA-UE, FIM | EMG-stim treatment led to superior upper-limb motor and functional outcomes compared with control therapy. |
| Murakami, 2023 | EG: 11 CG: 9 | EG: 50.4(9.8) CG: 49.8(11.4) | Chronic | 8 sessions,  2/week for 4 weeks | Artificial intelligence-integrated EMG driven robot hand (active) group | Passive robot | Yes (< 3mo) | FMA-UE, MAL (AOU), MAS (wrist and finger) | The Artificial intelligence-integrated EMG-driven robotic hand produced sustained improvements in upper-limb motor function and spasticity after stroke. |
| Wilson, 2016 | EG: 41 CG: 81 | EG: 58.6(48.9-67.5)* CG1: 55(47.4–65.9)*  CG2: 55.8(47.6-66.7)* | Subacute | 80 sessions,  10/week for 8 weeks | EMG-Triggered NMES | Cyclic NMES OR sensory stimulation  (combination of groups) | Yes (< 3mo and ≥ 3mo) | FMA-UE, AMAT | EMG-triggered NMES provided no additional benefit compared with non-triggered cyclic NMES or sensory stimulation. |
| Thielbar, 2017 | EG: 11 CG: 11 | EG: 61(12)  CG: 56(10) | Chronic | 18 sessions,  3/week for 6 weeks | Voice and electromyography driven actuated (VAEDA) glove + occupational therapy | Task-oriented occupational therapy | Yes (< 3mo) | FMA-UE, CMSA-H, WMFT (time), ARAT, Grip strength, Hand aperture | The VAEDA glove increased therapy effectiveness and shows promise as a clinical tool for improving hand function. |
| Cauraugh, 2000 | EG: 7 CG: 4 | 61.64(9.57) [Overall] | Chronic | 12 sessions,  6/week for 2 weeks | EMG-triggered NMES + conventional rehabilitation | Conventional rehabilitation | No | BBT, MAS, FMA-UE | EMG-triggered NMES significantly improved small-object grasping and sustained extensor contractions in individuals with chronic hemiparesis. |
| Dorsch, 2014 | EG: 16 CG: 17 | EG: 66(12) CG: 69 (13) | Acute | 20 sessions,  5/week for 4 weeks | EMG-triggered electrical stimulation + Conventional rehabilitation | Conventional rehabilitation | Yes (< 3mo) | MMT, Motor assessment scale | EMG-triggered stimulation is feasible in very weak early post-stroke patients but provides no added benefit over conventional arm therapy. |
| Kwakkel, 2016 | EG: 50 CG: 51 | EG: 58.94(11.64) CG: 58.53 (11.82) | Acute | 30 sessions,  10/week for 3 weeks | EMG-NMES | Conventional rehabilitation | Yes (< 3mo and ≥ 3mo) | ARAT, FMA-UE, Motricity index, EmNSA-UE, SIS, WMFT (time and FAS), FAT, MAL (AOU, QOM), NHPT | EMG-NMES treatment did not show significant benefits compared to conventional rehabilitation in patients with poor prognosis (no voluntary finger extension). |
| Kim, 2015 | EG: 10 CG: 19 | EG: 58.10(8.32) CG1: 53.20(7.91) CG2: 62.11(1.37) | Chronic | 20 sessions,  5/week for 4 weeks | EMG Biofeedback-functional electrical stimulation + Mirror training + conventional rehabilitation | Functional electrical estimulation + mirror therapy + conventional rehabilitation OR conventional rehabilitation | No | MMT, ROM (wrist flexion and extension), MAS (wrist), Grip strength, FIM, BBT, JTHFT, SSQOL | Mirror therapy combined with EMG biofeedback-functional electrical stimulation is a feasible and effective approach for upper-limb motor recovery after stroke. |
| Jonsdottir, 2017 | EG: 32 CG: 36 | EG: 68.3(36.5-84.8)*  CG: 67.7(38-88.7)* | Subacute/  Chronic | 25 sessions,  5/week for 5 to 6 weeks | Myoelectric control of functional electrical stimulation assisted task-oriented training + Physical therapy | Conventional rehabilitation including task-oriented training + Physical therapy | Yes (< 3mo) | ARAT, FMA-UE, DASH | Task-oriented arm rehabilitation improved function in all participants but adding myoelectric control of functional electrical stimulation led to better outcomes, especially in the subacute phase after stroke. |
| Hu, 2009 | EG: 15 CG: 12 | EG: 49.2(14.7) CG: 53.3(10.4) | Chronic | 20 sessions,  3 to 5/week for 7 weeks | EMG-driven robot assisted interactive wrist treatment | robotic device providing continuous passive movement | Yes (≥ 3mo) | FMA-UE, MAS (wrist), ARAT, FIM | Sustained improvements in coordination and spasticity were observed with the experimental treatment, while the control group showed only wrist flexor spasticity reduction. |
| * Data reported as median with min and max or interquartile ranges; NI: No information; FES: Functional Electrical Stimulation; NMES: Neuromuscular electrical stimulation; ARAT: Action Research Arm test; BFM: Brunnstrom Fugl-Meyer; FIM: Functional independence measure; SIS: Stroke Impact Scale; IPPA: Individually Prioritized Problem Assessment; FMA-UE: Fugl-Meyer Assessment Upper Extremity; MFT: Manual function test; ROM: Range of Motion; BI: Barthel Index; RPS: Reaching Performance Scale; BBT: Box and Block Test; MAS: Modified Ashworth Score; WMFT: Wolf Motor Function Test; FAS: Functional Ability Scale; MAL: Motor Activity Log; AOU: Amount of Use; QOM: Quality of Movement; MMDT: Minnesota Manual Dexterity Test; UEFT: Upper extremity function test; MSS: Motor status scale; JHFT: Jebsen Hand Function Test; MMT: Manual muscle test; AMAT: Arm Motor Ability Test; CMSA-H: ChedokeMcMaster Stroke Assessment – Hand scale; EmNSA-UE: Erasmus MC modified Nottingham Sensory Assessment of the upper extremity; FAT: Frenchay Arm Test; NHPT: Nine Hole Peg Test; JTHFT: Jebsen Taylor Hand Function Test; SSQOL: Stroke Specific Quality Of Life scale; DASH: Disability of the arm, shoulder and hand questionnaire. | | | | | | | | | |
