# Supplementary material for: Impact of EMG-based interfaces for hand motor rehabilitation in stroke: A systematic review with evidence gap map and meta-analysis
Source: Med Biol Eng Comput. 2026 Aug 28;64(10):3675–701. doi: 10.1007/s11517-026-03660-7 (PMC13723694; doi:10.1007/s11517-026-03660-7)
Supplement: Supplementary file 7 [file 11517_2026_3660_MOESM7_ESM.docx]

|  | Domain 1 | Domain 2 | Domain 3 | Domain 4 | Domain 5 | Overall |
| --- | --- | --- | --- | --- | --- | --- |
| Hemmen et al. 2007 |  |  |  |  |  |  |
| Unal et al. 2019 |  |  |  |  |  |  |
| Thorsen et al. 2013 |  |  |  |  |  |  |
| Kim et al. 2023 |  |  |  |  |  |  |
| Perini et al. 2021 |  |  |  |  |  |  |
| Qian et al. 2017 |  |  |  |  |  |  |
| Chen et al. 2022 |  |  |  |  |  |  |
| Armagan et al. 2003 |  |  |  |  |  |  |
| Lourenção et al. 2008 |  |  |  |  |  |  |
| Coskunsu et al. 2022 |  |  |  |  |  |  |
| Schick et al. 2022 |  |  |  |  |  |  |
| Jung et al. 2020 |  |  |  |  |  |  |
| Aslan et al. 2012 |  |  |  |  |  |  |
| Zhou et al. 2017 |  |  |  |  |  |  |
| de Kroon et al. 2008 |  |  |  |  |  |  |
| Shin et al. 2008 |  |  |  |  |  |  |
| Cauraugh et al. 2002 |  |  |  |  |  |  |
| Habib et al. 2020 |  |  |  |  |  |  |
| Park et al. 2017 |  |  |  |  |  |  |
| Kim et al. 2017 |  |  |  |  |  |  |
| Rayegani et al. 2014 |  |  |  |  |  |  |
| Amasyali et al. 2016 |  |  |  |  |  |  |
| Kojima et al. 2014 |  |  |  |  |  |  |
| Gabr et al. 2005 |  |  |  |  |  |  |
| Boyaci et al. 2013 |  |  |  |  |  |  |
| Zhao et al. 2022 |  |  |  |  |  |  |
| Heckmann et al. 1997 |  |  |  |  |  |  |
| Hong et al. 2012 |  |  |  |  |  |  |
| Francisco et al. 1998 |  |  |  |  |  |  |
| Murakami et al. 2023 |  |  |  |  |  |  |
| Wilson et al. 2016 |  |  |  |  |  |  |
| Thielbar et al. 2017 |  |  |  |  |  |  |
| Cauraugh et al. 2000 |  |  |  |  |  |  |
| Dorsch et al. 2014 |  |  |  |  |  |  |
| Kwakkel et al. 2016 |  |  |  |  |  |  |
| Kim et al. 2015 |  |  |  |  |  |  |
| Jonsdottir et al. 2017 |  |  |  |  |  |  |
| Hu et al. 2009 |  |  |  |  |  |  |

# Supplementary file 7. Risk of Bias by each included study

Low risk

Some concerns

High risk

Domain 1: Randomization Process

Domain 2: Deviations from intended interventions

Domain 3: Missing outcome data

Domain 4: Measurement of the reported result

Domain 5: Selection of the reported result
