# Supplementary material for: Impact of EMG-based interfaces for hand motor rehabilitation in stroke: A systematic review with evidence gap map and meta-analysis
Source: Med Biol Eng Comput. 2026 Aug 28;64(10):3675–701. doi: 10.1007/s11517-026-03660-7 (PMC13723694; doi:10.1007/s11517-026-03660-7)
Supplement: Supplementary file 8 [file 11517_2026_3660_MOESM8_ESM.docx]

# Supplementary file 8. Meta-analyses

### Symbols:

***** To studies including more comparators

**+** To studies including only CT or Mirror therapy

**^** To studies including other control interventions

## 1. Additional meta-analyses of EMG-ES combined with other interventions

Figure 1. Meta-analysis of the effects of EMG-ES combined with other interventions versus the comparators on the body functions and structures domain of the International Classification of Functioning, Disability and Health (ICF), assessed using Fugl-Meyer Assessment – wrist (FMA-Wrist) immediately after intervention. Abbreviations: CT, conventional therapy; ES, electrical stimulation.

Figure 2. Meta-analysis of the effects of EMG-ES combined with other interventions versus the comparators on the body functions and structures domain of the International Classification of Functioning, Disability and Health (ICF), assessed using Fugl-Meyer Assessment – hand (FMA-Hand) immediately after intervention. Abbreviations: CT, conventional therapy; ES, electrical stimulation.

Figure 3. Meta-analysis of the effects of EMG-ES combined with other interventions versus the comparators on the body functions and structures domain of the International Classification of Functioning, Disability and Health (ICF), assessed using Fugl-Meyer Assessment – coordination/speed (FMA-Coor/Speed) immediately after intervention. Abbreviations: CT, conventional therapy; ES, electrical stimulation.

Figure 4. Meta-analysis of the effects of EMG-ES combined with other interventions versus the comparators on the body functions and structures domain of the International Classification of Functioning, Disability and Health (ICF), assessed using Modified Ashworth Scale – wrist flexor (MAS – Wrist flexor) immediately after intervention. Abbreviations: CT, conventional therapy; ES, electrical stimulation.

## 2. Sensitivity analysis - EMG-ES combined with other interventions

Figure 5. Sensitivity analysis excluding Shin et al. 2008 of the effects of EMG-ES combined with other interventions versus the comparators on the activity domain of the International Classification of Functioning, Disability and Health (ICF), assessed using Box and Block Test (BBT) immediately after intervention. Abbreviations: CT, conventional therapy; Th, Therapy; ES, electrical stimulation.

## 3. Effects of EMG-based robotic platforms

Figure 6. Meta-analysis of the effects of EMG-based robotic platforms versus control interventions on the body functions and structures domain of the International Classification of Functioning, Disability and Health (ICF), assessed using the Fugl-Meyer Assessment – Upper Extremity (FMA-UE) immediately after intervention (A) and at follow-up ≥ 3 months (B), and using the Wolf Motor Function Test (WMFT)-Time immediately after intervention (C). Abbreviations: CT, conventional therapy.

Figure 7. Meta-analysis of the effects of EMG-based robotic platforms versus control interventions on the activity domain of the International Classification of Functioning, Disability and Health (ICF), assessed using the Action Research Arm Test (ARAT) immediately after intervention (A) and at follow-up ≥ 3 months (B). Abbreviations: CT, conventional therapy.

Figure 8. Meta-analysis of the effects of EMG-based robotic platforms versus control interventions on the participation and patient-reported outcomes domains of the International Classification of Functioning, Disability and Health (ICF), assessed using the Functional Independence Measure (FIM) immediately after intervention (A) and at follow-up ≥ 3 months (B), and using Motor Activity Log – Amount of Use (MAL-AOU) (C) and Motor Activity Log – Quality of Movement (MAL-QOM) (D) at immediately after intervention. Abbreviations: CT, conventional therapy.

Figure 9. Meta-analysis of the effects of EMG-based robotic platforms versus control interventions on the body functions and structures domain of the International Classification of Functioning, Disability and Health (ICF), assessed using Fugl-Meyer Assessment – wrist (FMA-Wrist) immediately after intervention. Abbreviations: CT, conventional therapy.

Figure 10. Meta-analysis of the effects of EMG-based robotic platforms versus control interventions on the body functions and structures domain of the International Classification of Functioning, Disability and Health (ICF), assessed using Fugl-Meyer Assessment – hand (FMA-Hand) immediately after intervention. Abbreviations: CT, conventional therapy.

Figure 11. Meta-analysis of the effects of EMG-based robotic platforms versus control interventions on the body functions and structures domain of the International Classification of Functioning, Disability and Health (ICF), assessed using Fugl-Meyer Assessment – coordination/speed (FMA-Coor/Speed) immediately after intervention. Abbreviations: CT, conventional therapy.

Figure 12. Meta-analysis of the effects of EMG-based robotic platforms versus control interventions on the body functions and structures domain of the International Classification of Functioning, Disability and Health (ICF), assessed using Fugl-Meyer Assessment – hand (FMA-Hand) at follow-up < 3 months. Abbreviations: CT, conventional therapy.

Figure 13. Meta-analysis of the effects of EMG-based robotic platforms versus control interventions on the body functions and structures domain of the International Classification of Functioning, Disability and Health (ICF), assessed using Fugl-Meyer Assessment – wrist/hand (FMA-Wrist/hand) immediately after intervention.

 Figure 14. Meta-analysis of the effects of EMG-based robotic platforms versus control interventions on the body functions and structures domain of the International Classification of Functioning, Disability and Health (ICF), assessed using Fugl-Meyer Assessment – wrist/hand (FMA-Wrist/hand) at follow-up ≥ 3 months.

Figure 15. Meta-analysis of the effects of EMG-based robotic platforms versus control interventions on the body functions and structures domain of the International Classification of Functioning, Disability and Health (ICF), assessed using Modified Ashworth Scale – finger (MAS – Finger) immediately after intervention.

Figure 16. Meta-analysis of the effects of EMG-based robotic platforms versus control interventions on the body functions and structures domain of the International Classification of Functioning, Disability and Health (ICF), assessed using Modified Ashworth Scale – wrist (MAS – Wrist) immediately after intervention.

Figure 17. Meta-analysis of the effects of EMG-based robotic platforms versus control interventions on the body functions and structures domain of the International Classification of Functioning, Disability and Health (ICF), assessed using Modified Ashworth Scale – wrist (MAS – Wrist) at follow-up ≥ 3 months.

### Control group symbols:

a) Task-oriented intervention
b) Robotic device providing continuous passive movement
c) Passive robot
d) Conventional therapy

## 4. Effects of EMG biofeedback combined with other interventions

Figure 18. Meta-analysis of the effects of EMG biofeedback combined with other interventions versus comparators on the body functions and structures domain of the International Classification of Functioning, Disability and Health (ICF), assessed using wrist extension Range of Motion (ROM) (A), and Brunnstrom score (B). Both were evaluated immediately after the intervention. Abbreviations: BF, biofeedback; CT, conventional therapy; FES, functional electrical stimulation.
